# Magnesium in Skin Aging, Wound Healing, and Some Specific Dermatologic Disorders: A Narrative Review on Biological Mechanisms, Clinical Evidence, and Therapeutic Perspectives

**DOI:** 10.3390/nu18213583

**Published:** 2026-10-29

**Authors:** Ligia J. Dominguez, Valentina Caputo, Francesco Saverio Ragusa, Nicola Veronese, José Luis Pérez-Albela, Vania Flora Vásquez-López, Santiago Rodas-Regalado, Mario Barbagallo

**Affiliations:** 1 Department of Psychology, Educational Science and Human Movement, University of Palermo, 90100 Palermo, Italy; 2 Pro.M.I.S.E. Department “Giuseppe D’Alessandro”, University of Palermo, 90100 Palermo, Italy; valentina.caputo@unipa.it (V.C.); francescosaverioragusa@gmail.com (F.S.R.); mario.barbagallo@unipa.it (M.B.); 3 Section of Dermatology, Department of Internal Medicine and Geriatrics, *“Paolo Giaccone”* University Hospital, 90100 Palermo, Italy; 4 Geriatric Unit, Department of Internal Medicine and Geriatrics, *“Paolo Giaccone”* University Hospital, 90100 Palermo, Italy; 5 Faculty of Medicine, Saint Camillus International University of Health Sciences, 00131 Rome, Italy; nicola.veronese@unicamillus.org; 6 Instituto Bien de Salud, Lima 15036, Peru; joseluisperezalbela@gmail.com (J.L.P.-A.); vaniaelam2009@gmail.com (V.F.V.-L.); santir775@gmail.com (S.R.-R.)

**Keywords:** magnesium, dermatology, skin, atopic dermatitis, psoriasis, acne vulgaris, wound healing, oxidative stress, inflammation, immune regulation, nutritional dermatology, skin aging

## Abstract

Magnesium is the second most abundant intracellular cation and an essential cofactor for more than 600 enzymatic reactions involved in cellular metabolism, energy production, nucleic acid synthesis, protein translation, and immune regulation. Emerging experimental evidence suggests that magnesium contributes to several processes involved in skin homeostasis, including keratinocyte proliferation and differentiation, epidermal barrier integrity, collagen synthesis, oxidative balance, and innate and adaptive immune responses. Magnesium deficiency is common but often underrecognized, particularly among persons at higher risk including older adults and persons with gastrointestinal disorders, type 2 diabetes, chronic alcohol use, or certain medication exposures. This deficiency has been implicated in the pathogenesis and progression of several dermatologic diseases through mechanisms involving chronic inflammation, oxidative stress, impaired wound repair, endothelial dysfunction, and immune dysregulation. This review summarizes the current understanding of magnesium biology in skin physiology and critically examines experimental and clinical evidence linking altered magnesium status to inflammatory, autoimmune, and degenerative skin disorders, including atopic dermatitis, psoriasis, acne vulgaris, rosacea, chronic urticaria, wound healing disorders, diabetic foot ulcers, alopecia, and skin aging. The review also discusses the therapeutic potential of oral and topical magnesium formulations, including their proposed mechanisms of action, clinical efficacy, safety profile, and current limitations of the available evidence. Although preclinical studies support a role for magnesium in skin health, clinical evidence remains limited by heterogeneous study designs and a lack of high-quality randomized trials. Future research should prioritize standardized biomarkers and robust clinical studies to clarify the therapeutic potential of magnesium and identify patients most likely to benefit from magnesium-based interventions in dermatologic disorders.

## 1. Introduction

Magnesium is the second most abundant intracellular cation and the fourth most abundant cation in the human body. It is an essential micronutrient that serves as a cofactor for more than 600 enzymatic reactions involved in energy metabolism, nucleic acid and protein synthesis, ion transport, cell signaling, and maintenance of cellular homeostasis [1,2,3]. Approximately 50–60% of total body magnesium is stored in bone, mostly associated with the hydroxyapatite mineral phase, while the remainder is found in intracellular soft tissues, particularly skeletal muscle, with only 1–2% present in extracellular fluids, making assessment of magnesium status particularly challenging [1,2,3,4].

Magnesium deficiency is a common but frequently underrecognized clinical condition [5,6,7]. Magnesium homeostasis is tightly regulated through coordinated intestinal absorption, renal excretion, and bone exchange. Specialized transport proteins, including transient receptor potential melastatin (TRPM) channels, particularly TRPM6 and TRPM7, play fundamental roles in maintaining intracellular magnesium concentrations and regulating numerous physiological processes [1,8]. Despite these regulatory mechanisms, inadequate dietary intake, gastrointestinal disorders, chronic kidney disease, diabetes mellitus, medication use (e.g., diuretics, proton pump inhibitors, antineoplastic drugs), alcohol abuse, and aging contribute to a high prevalence of subclinical magnesium deficiency worldwide, which has become an increasingly recognized public health concern [2,9,10,11,12,13]. It has been estimated that hypomagnesemia affects 3–10% of the general population, 10–30% of individuals with type 2 diabetes, and 10–60% of hospitalized patients, rising to over 65% in intensive care units [14].

In recent years, increasing attention has focused on the role of magnesium beyond its classical functions in neuromuscular and cardiovascular physiology. A growing body of experimental evidence indicates that magnesium is a critical regulator of inflammatory responses, oxidative stress, mitochondrial function, endothelial integrity, and both innate and adaptive immunity [15,16], all of which are fundamental processes involved in skin homeostasis and the pathogenesis of inflammatory dermatologic diseases. Magnesium deficiency has been associated with increased production of pro-inflammatory cytokines, activation of nuclear factor-κB (NF-κB), enhanced oxidative stress, endothelial dysfunction, and impaired tissue repair [15,16,17,18], suggesting that inadequate magnesium availability may contribute to chronic inflammatory conditions affecting multiple organ systems, including the skin.

The skin is a highly dynamic organ that requires tightly coordinated cellular proliferation, differentiation, immune surveillance, and extracellular matrix remodeling to preserve barrier integrity and maintain tissue homeostasis. Magnesium has been implicated in several processes involved in skin homeostasis and repair, including keratinocyte proliferation and migration, fibroblast activity, extracellular-matrix remodeling and collagen deposition, epidermal barrier repair, and cutaneous wound healing [19,20,21,22,23].

Moreover, magnesium plays an important role in calcium and cellular signaling, ATP-dependent enzymatic reactions, and mitochondrial function, while magnesium status can influence cellular antioxidant defenses [3,4,8,16,24,25]. These functions contribute to epidermal homeostasis and may help protect keratinocytes against oxidative stress, including UV-induced injury [26,27].

Accumulating experimental and clinical studies have suggested that disturbances in magnesium homeostasis may be involved in the pathophysiology of several dermatologic disorders. Altered magnesium status has been reported in several inflammatory and inflammatory-associated skin conditions, particularly atopic dermatitis, psoriasis, and acne vulgaris, although findings are inconsistent across studies. More consistent evidence of lower magnesium levels is available in patients with diabetic foot ulcers, where observational studies and meta-analytic evidence have demonstrated reduced serum magnesium compared with diabetic and healthy controls [28,29,30,31,32]. Furthermore, topical magnesium-containing formulations, including magnesium-rich mineral baths and Dead Sea salt preparations, have demonstrated beneficial effects on skin hydration, barrier function, and symptom relief in selected inflammatory dermatoses, while oral magnesium supplementation has shown promise in improving inflammatory and metabolic parameters that may indirectly influence disease severity [17,33,34,35,36].

Despite increasing interest in the dermatological implications of magnesium, current evidence remains fragmented. Many available studies are limited by small sample sizes, heterogeneous patient populations, inconsistent methods for assessing magnesium status, and variability in supplementation regimens, making it difficult to establish definitive clinical recommendations. Consequently, the precise role of magnesium deficiency as a causal factor, biomarker, or therapeutic target in dermatologic disease remains incompletely understood.

The purpose of this narrative review is to provide a comprehensive overview of the biological functions of magnesium relevant to skin physiology, summarize current evidence linking magnesium homeostasis to inflammatory, autoimmune, and degenerative skin disorders, critically evaluate the therapeutic potential of oral and topical magnesium supplementation, and identify key knowledge gaps and future research priorities. A clearer understanding of magnesium biology in dermatology may facilitate the development of evidence-based nutritional and pharmacological strategies to improve the prevention and management of a wide range of cutaneous diseases.

## 2. Search Methods

For this narrative review, PubMed/MEDLINE was searched for English-language peer-reviewed articles published from the database’s inception to July 1st, 2026. The search combined terms related to magnesium and skin disorders. The principal search strategy was (‘magnesium’) AND (‘dermatology’, OR ‘skin’, OR ‘skin disorder’, OR ‘skin disease’, OR ‘atopic dermatitis’, OR ‘psoriasis’, OR ‘acne’, OR ‘urticaria’, OR ‘wound healing’, OR ‘foot ulcer’, OR ‘oxidative stress’, OR ‘inflammation’, OR ‘immune regulation’, OR ‘hair disorders’ OR ‘nail disorders’, OR ‘skin aging’). Longitudinal studies, randomized or non-randomized clinical studies, narrative and systematic reviews, meta-analyses, umbrella reviews, consensus statements, and clinical guidelines were considered. Reference lists of relevant publications were hand-searched, and forward citation searching was used to identify additional pertinent studies. Earlier landmark publications were retained when required for historical definitions, diagnostic criteria, or foundational evidence. Case reports, case series, editorials, letters, comments, and short communications were excluded. Because this was a narrative rather than systematic review, no formal risk-of-bias assessment or quantitative evidence synthesis was performed. Emphasis was placed on the largest comprehensive studies and the most recent publications.

## 3. Magnesium Biology

### 3.1. Physiological Function and Homeostasis

Magnesium is a physiologically essential mineral, the second most abundant intracellular cation, and the most abundant intracellular divalent cation. It serves as a critical cofactor for numerous enzymatic reactions and is required for oxidative phosphorylation, energy production, protein and nucleic acid synthesis, and glycolysis [3,37,38,39]. Magnesium also facilitates ion transport across cell membranes and contributes to muscle contraction, cardiac rhythm, and neuronal excitability [2,40,41]. Furthermore, it forms MgATP, the biologically active form of ATP, which is essential for cellular energy metabolism and signaling processes, including protein phosphorylation, cAMP activation, gene expression, DNA synthesis, glucose metabolism, and neuromuscular function [3,8,37,39,41]. Magnesium is predominantly intracellular (~98%), while only ~1–2% circulates in serum. Serum magnesium is tightly regulated through a dynamic balance between dietary intake, intestinal absorption, renal excretion, bone storage, and tissue requirements [3,38,39,41]. Approximately 30–50% of dietary magnesium is absorbed, primarily in the small intestine through both passive paracellular pathways and active transcellular transport involving TRPM6 and TRPM7 channels [1,8,42]. TRPM6 is highly expressed in the distal convoluted tubule of the kidney and in the intestine, where it contributes to the regulation of systemic magnesium balance, whereas TRPM7 is ubiquitously expressed and participates in cellular magnesium and calcium homeostasis. Both proteins possess channel and kinase domains, enabling them to integrate ion transport with intracellular signaling and cellular regulatory mechanisms. Through these functions, TRPM6 and TRPM7 contribute to essential physiological processes, including enzymatic activity, energy metabolism, protein synthesis, cell proliferation, differentiation, and signal transduction. In the skin, these mechanisms are of particular interest because keratinocyte proliferation and differentiation, epidermal lipid synthesis, and maintenance of the permeability barrier depend on tightly regulated cellular processes. Nevertheless, although magnesium transport provides a plausible mechanistic link to epidermal homeostasis, the specific contribution of TRPM6 and TRPM7 dysfunction to the pathogenesis of inflammatory or hyperproliferative skin disorders remains incompletely understood and warrants further investigation.

The kidneys are the principal regulators of circulating magnesium: most filtered magnesium is reabsorbed along the nephron, with substantial reabsorption occurring in the thick ascending limb and fine regulation in the distal convoluted tubule, particularly through TRPM6 [8,42]. Bone represents the major magnesium reservoir and can contribute to maintaining extracellular magnesium concentrations when intake is insufficient [43]. Overall, changes in dietary intake and body magnesium stores are compensated by coordinated adjustments in intestinal absorption and renal magnesium handling, thereby maintaining relatively stable plasma magnesium concentrations [42]. 

### 3.2. Assessment of Magnesium Status

Assessing magnesium status is challenging because, as mentioned, only ~1–2% of total body magnesium is present in extracellular fluid, while most is stored intracellularly and in bone; therefore, no single biomarker reliably reflects total-body magnesium status [44]. 

Total serum magnesium is the most widely used and accessible clinical measure. It is useful for detecting overt hypomagnesemia and acute changes in extracellular magnesium, but it has limited sensitivity for subclinical or intracellular magnesium deficiency because serum concentrations are tightly regulated and represent only a small fraction of total-body magnesium. Therefore, normal serum magnesium levels may not exclude intracellular magnesium deficiency [45]. Serum magnesium is also influenced by albumin, renal function, acid–base status, and other circulating ligands [46]. Ionized magnesium represents the biologically active, unbound fraction of circulating magnesium and may provide greater physiological information than total serum magnesium. Approximately 60–70% of circulating magnesium is ionized, with the remainder predominantly protein-bound or complexed to anions. However, ionized magnesium is not routinely available, and methodological and pre-analytical differences limit its standardization and clinical application [45,47]. Because magnesium is predominantly intracellular, red blood cells (RBC) magnesium has been proposed as a more representative measure of intracellular magnesium status than serum magnesium. Nevertheless, its clinical utility remains uncertain because of substantial biological variability, differences in analytical methods, RBC age, supplementation, potential sample hemolysis, and the lack of standardized reference ranges. In addition, RBC magnesium testing is not routinely available in clinical practice [9]. 

Twenty-four-hour urinary magnesium excretion provides information about recent magnesium intake and renal magnesium handling and may be useful when interpreted together with serum magnesium and dietary intake. Magnesium excretion generally decreases when dietary intake is restricted, reflecting renal conservation. However, urinary magnesium is influenced by dietary intake, renal function, medications, and hormonal regulation, and therefore does not independently provide a reliable estimate of total-body magnesium status [48]. The magnesium loading or tolerance test assesses magnesium status by measuring urinary magnesium excretion over 24 h following an intravenous or oral magnesium load. Magnesium-deficient individuals retain a greater proportion of the administered magnesium, whereas magnesium-replete individuals excrete most of the load. Thus, the percentage of magnesium retained provides an estimate of whole-body magnesium status and may correlate with tissue, including bone, magnesium stores. However, the test is influenced by renal function and gastrointestinal absorption and is not routinely used in clinical practice [46].

Thus, the major limitation is the absence of a simple, standardized biomarker that accurately reflects whole-body and intracellular magnesium stores. A systematic review found that serum/plasma magnesium, RBC magnesium, and urinary magnesium respond to dietary manipulation, but concluded that evidence remains insufficient to establish a single optimal biomarker [49]. Current recommendations therefore favor integrating serum magnesium with urinary magnesium, dietary intake, clinical risk factors, and, when appropriate, functional tests such as the magnesium-loading/tolerance test rather than relying on one measurement alone [48]. A recent 2026 review similarly concluded that no single biomarker reliably reflects whole-body magnesium status and that combined approaches are likely to provide better assessment [44]. Table 1 summarizes the main methods used to assess magnesium status.

## 4. Magnesium and Skin Physiology

**Figure 1:**
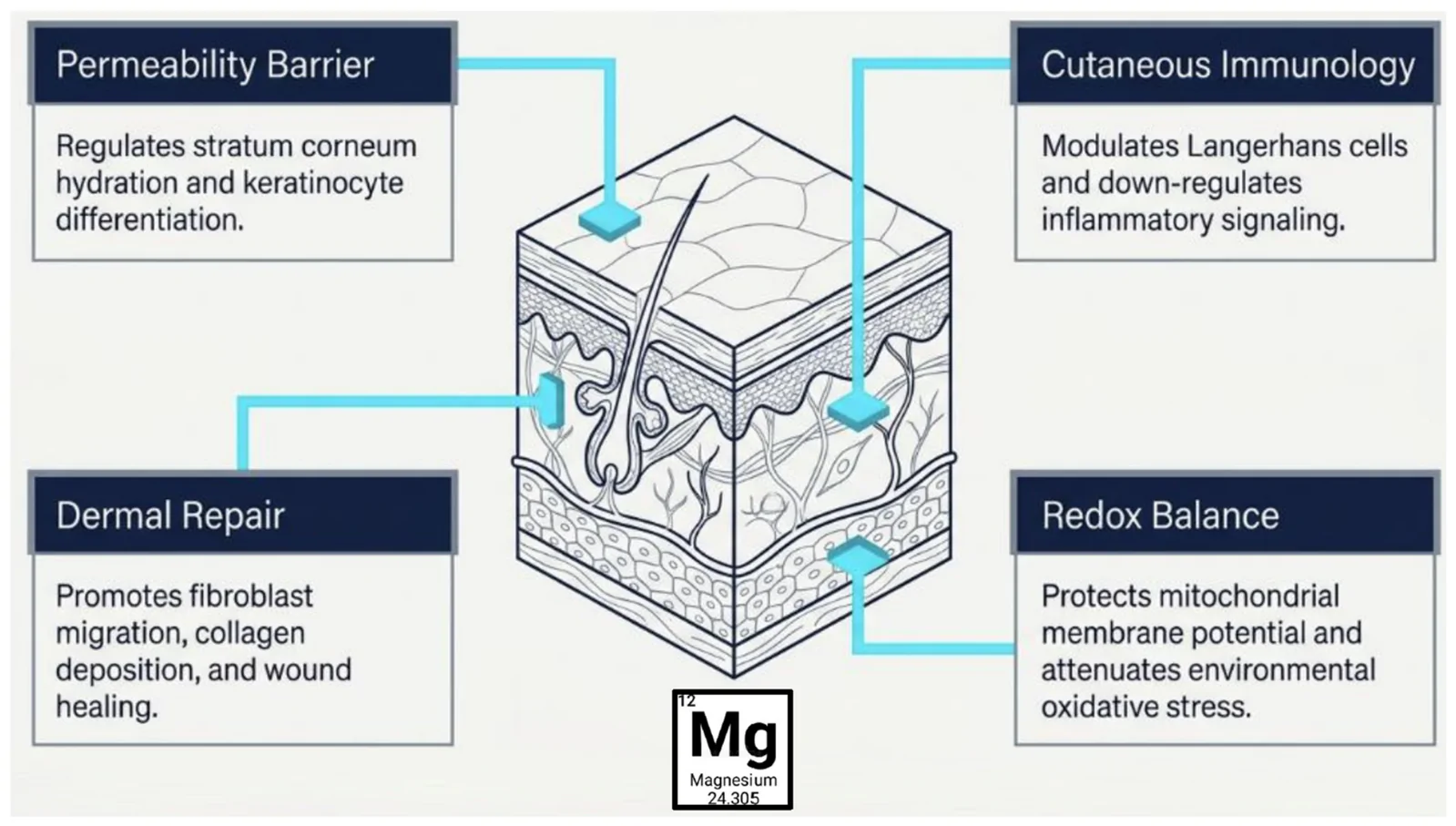
Summary of relevant magnesium actions on skin physiology.

Magnesium is an essential mineral involved in numerous cellular processes that contribute to skin homeostasis, including enzymatic activity, protein synthesis, cellular energy metabolism, and redox regulation. In skin physiology, magnesium may support epidermal barrier integrity, keratinocyte differentiation, dermal repair, and cutaneous immune regulation. By contributing to cellular stress responses and maintaining physiological balance, magnesium may help preserve normal skin function (Figure 1). However, its effects depend on its availability, the route of exposure, and the specific physiological context.

### 4.1. Epidermal Homeostasis

Magnesium appears to contribute to epidermal homeostasis by influencing keratinocyte proliferation, differentiation, migration, and epidermal barrier function. Magnesium is present in the epidermis and, together with calcium, participates in ionic gradients and signaling processes that regulate keratinocyte behavior and the formation and repair of the stratum corneum [53]. Experimental studies indicate that extracellular magnesium can modulate keratinocyte growth. Increased magnesium concentrations stimulated proliferation and DNA synthesis in cultured mouse epidermal keratinocytes, although magnesium alone did not induce differentiation [19]. More broadly, magnesium and calcium gradients appear to interact in regulating keratinocyte differentiation and epidermal maturation, suggesting that the calcium/magnesium balance, rather than magnesium concentration alone, may be important for epidermal homeostasis [21]. 

Magnesium has also been implicated in maintaining and restoring the permeability barrier. In animal models, topical magnesium salts accelerated epidermal barrier recovery after disruption, with optimal repair associated with a favorable calcium/magnesium ratio [21]. 

Magnesium may additionally support epidermal integrity by influencing cellular energy metabolism and responses to oxidative stress. In human keratinocytes, intracellular magnesium responses to hydrogen peroxide were associated with preservation of mitochondrial membrane potential, while magnesium supplementation reduced oxidative-stress-induced cell death [26]. Similarly, MgCl_2_ supplementation attenuated UVB-induced reactive oxygen species (ROS) and improved keratinocyte viability in an experimental model [27]. 

Overall, available evidence suggests that magnesium contributes to epidermal homeostasis through interactions with calcium signaling, keratinocyte proliferation and differentiation, barrier repair, and cellular responses to oxidative stress. However, much of the mechanistic evidence remains experimental, and the specific contribution of magnesium to human epidermal physiology requires further clinical investigation [54]. 

### 4.2. Dermal Functions

Magnesium may contribute to dermal homeostasis and tissue repair through effects on fibroblasts, extracellular-matrix (ECM) remodeling, collagen organization, angiogenesis, and inflammatory signaling. In the dermis, these processes are essential for maintaining connective-tissue integrity and coordinating wound repair. In a hydrogel model, sustained magnesium release enhanced human dermal fibroblast migration and myofibroblast differentiation, and increased collagen deposition and angiogenesis during wound healing in rats [22].

Magnesium may also influence collagen synthesis and structural organization. Experimental studies have demonstrated effects of magnesium-containing compounds on collagen synthesis in human dermal fibroblasts, while magnesium-modified collagen matrices have been shown to alter collagen structure and fibroblast behavior [55,56]. However, evidence involving magnesium ascorbyl phosphate should be interpreted cautiously because the biological effects cannot necessarily be attributed to magnesium alone. Magnesium also appears to support cutaneous wound healing. Topical magnesium treatment accelerated healing in a rat burn model, increasing fibroblast numbers, collagen volume, vascularization, and tissue strength while reducing TNF-α and malondialdehyde levels [23]. Similarly, experimental magnesium-containing biomaterials have promoted collagen deposition, angiogenesis, re-epithelialization, and ECM remodeling [22]. These findings suggest that magnesium may influence several phases of dermal repair, including inflammation, proliferation, ECM formation, and tissue remodeling.

Overall, current evidence indicates that magnesium contributes to dermal functions by modulating fibroblast activity, collagen/ECM organization, angiogenesis, and inflammatory responses, with potential relevance to wound healing and tissue regeneration. Nevertheless, much of the available evidence remains preclinical, and further human studies are needed to establish the clinical effects of magnesium on normal dermal physiology and wound repair [57]. 

### 4.3. Skin Immunological Functions

Magnesium appears to play an important role in cutaneous immune homeostasis, influencing inflammatory signaling, antigen presentation, oxidative responses, and the activity of immune cells within the skin. Magnesium deficiency has been associated with enhanced inflammatory responses, including increased cytokine production, oxidative stress, and activation of innate immune cells. These effects are thought to involve altered calcium signaling, redox imbalance, and dysregulation of magnesium transporters [15,16,17,18].

Within the skin, magnesium may directly modulate epidermal immune surveillance. Experimental studies have shown that MgCl_2_ can reduce the antigen-presenting capacity of Langerhans cells, accompanied by decreased expression of HLA-DR and B7 costimulatory molecules and reduced TNF-α production. These findings suggest that magnesium may help regulate excessive cutaneous immune activation and inflammatory responses [58]. In addition, magnesium may contribute to cutaneous responses to oxidative and environmental stress. In cultured human keratinocytes, MgCl_2_ supplementation reduced cellular damage following UVB exposure and increased polyamine production through MEK/GSK3/CREB-related signaling, suggesting a potential role for magnesium in protecting epidermal cells from UV-induced stress [27]. 

Overall, available evidence indicates that magnesium may support skin immune homeostasis by regulating Langerhans-cell activity, inflammatory cytokine signaling, oxidative stress, and keratinocyte responses. Nevertheless, much of the mechanistic evidence is experimental, and further clinical studies are required to determine the extent to which magnesium status or supplementation modifies human immune-mediated skin diseases [57]. 

### 4.4. Skin Oxidative Stress and Inflammation

Magnesium may contribute to cutaneous redox and inflammatory homeostasis by regulating cellular signaling, mitochondrial function, and immune responses. Magnesium deficiency has been associated with increased oxidative stress, characterized by greater lipid, protein, and DNA oxidation and impaired antioxidant defenses. These effects may be mediated partly through mitochondrial dysfunction, altered calcium homeostasis, and increased inflammatory signaling [59]. In the inflammatory response, low magnesium status can enhance activation of leukocytes and macrophages, increase production of pro-inflammatory cytokines such as TNF-α, IL-1, and IL-6, and promote oxidative burst and ROS generation. Magnesium may therefore act as a modulator of the relationship between inflammation and oxidative stress, with deficiency potentially creating a self-amplifying cycle in which inflammation increases ROS production and oxidative stress further promotes tissue injury [15]. Evidence specifically involving skin cells supports a potential protective role of magnesium against environmental oxidative stress. In cultured human HaCaT keratinocytes, MgCl_2_ supplementation reduced UVB-induced ROS accumulation and cell damage, an effect associated with increased polyamine synthesis through MEK/GSK3/CREB signaling. These findings suggest that magnesium may enhance keratinocyte resistance to UV-induced oxidative injury [27]. 

Magnesium may also influence cutaneous inflammation through regulation of immune-cell activity and inflammatory mediators. Experimental evidence indicates that magnesium can modulate antigen-presenting cells and inflammatory signaling, while magnesium-rich preparations have shown anti-inflammatory effects in some experimental and clinical skin studies. However, the dermatological evidence remains relatively limited, and many findings derive from in vitro or preclinical models rather than large clinical trials [60]. 

Overall, current evidence supports a potential role for magnesium in maintaining skin redox balance and controlling inflammatory responses, particularly by modulating ROS generation, antioxidant defenses, calcium-dependent signaling, cytokine production, and keratinocyte responses to environmental stress. Nevertheless, the evidence for a direct clinical benefit of magnesium supplementation in inflammatory or oxidative skin disorders remains preliminary. 

A recent meta-analysis involving persons with various health conditions, including coronary heart disease, diabetes, and polycystic ovary syndrome, reported that magnesium supplementation significantly reduced C-reactive protein (CRP) levels. However, its overall effects on oxidative stress biomarkers remained inconclusive, underscoring the need for further well-designed clinical studies [61]. 

### 4.5. Skin Barrier Dysfunction

Magnesium may contribute to epidermal barrier homeostasis through its effects on keratinocyte function, ionic balance, and barrier repair. Magnesium is present in the epidermis and interacts with calcium-dependent signaling pathways involved in keratinocyte proliferation and differentiation, processes that are essential for the formation and maintenance of the stratum corneum and epidermal permeability barrier [29]. 

Experimental evidence suggests that the Ca^2+^/Mg^2+^ balance may be particularly important for barrier recovery. Changes in epidermal ion gradients following barrier disruption regulate keratinocyte differentiation and the restoration of lipid and protein components of the barrier. Magnesium has been proposed to support these processes, although the specific contribution of magnesium remains less well established than that of calcium [62]. More broadly, magnesium may contribute to barrier protection by supporting cellular energy metabolism, keratinocyte differentiation, and responses to environmental stress. However, the current evidence should be interpreted cautiously because many mechanistic findings are derived from experimental models, while clinical studies frequently use magnesium-containing preparations with other minerals or active ingredients. 

### 4.6. Skin Microvascular Function

Magnesium may contribute to cutaneous microvascular homeostasis through effects on endothelial-cell function, vascular tone, inflammation, and angiogenesis. Experimental studies in microvascular endothelial cells show that magnesium availability directly influences endothelial proliferation and migration. Low magnesium concentrations inhibit endothelial growth and migration and increase inflammatory mediators such as IL-1α, IL-6, nitric oxide, and VCAM-1, whereas higher magnesium concentrations promote endothelial proliferation and migration, supporting a potential role in angiogenesis and microvascular repair [63,64]. Magnesium may also regulate vascular tone and endothelial-dependent vasodilation. It can act as a physiological calcium antagonist and influence nitric-oxide-dependent pathways, thereby promoting vascular smooth-muscle relaxation and vasodilation. These mechanisms could contribute to adequate tissue perfusion and oxygen delivery within the skin [65].

Clinical evidence for a systemic effect of magnesium on vascular function is mixed. A meta-analysis of seven randomized controlled trials involving 306 participants reported that magnesium supplementation significantly increased flow-mediated dilation (FMD), suggesting improved endothelial function, although substantial heterogeneity was observed between studies [66]. Conversely, another meta-analysis found no overall significant effect of oral magnesium on FMD or pulse-wave velocity, although benefits were observed in some subgroups, particularly older, overweight, or metabolically unhealthy individuals [67]. These findings may be indirectly relevant to skin microvascular function, as cutaneous perfusion depends on endothelial integrity, vascular smooth muscle activity, and microvascular reactivity. However, FMD primarily assesses conduit artery endothelial function, and pulse-wave velocity reflects arterial stiffness; neither directly measures cutaneous microcirculation. Therefore, the extent to which systemic vascular effects of magnesium translate into improved skin microvascular perfusion, tissue oxygenation, or wound healing remains uncertain and requires dedicated clinical investigation.

Importantly, direct evidence specifically examining magnesium and human skin microcirculation remains limited. A randomized controlled trial of magnesium supplementation in overweight/obese adults found no significant changes in retinal microvascular caliber or circulating markers of microvascular endothelial function after 24 weeks [68]. Therefore, although experimental evidence provides a plausible mechanism linking magnesium to cutaneous microvascular function, its specific effects on skin blood flow, angiogenesis, and microvascular reactivity in humans require further investigation.

### 4.7. Calcium–Magnesium Interactions

Calcium is a well-established regulator of keratinocyte differentiation, cell–cell adhesion, and epidermal barrier formation, while magnesium appears to modulate several of these calcium-dependent processes [62,69]. Experimental work in hairless mice showed that topical magnesium salts accelerated barrier recovery and that selected combinations of MgCl2 and CaCl2 produced greater recovery than MgCl2 alone, supporting the concept that the local Ca2+/Mg2+ balance may be relevant to epidermal repair [70].

However, the clinical importance of this interaction remains uncertain. Magnesium-rich Dead Sea salt bathing has improved hydration and transepidermal water loss (TEWL) in atopic dry skin, but the preparation contains multiple minerals and does not isolate a magnesium–calcium effect [33]. Thus, calcium and magnesium likely act in concert in epidermal physiology, but the optimal Ca2+/Mg2+ ratio in human skin and its therapeutic implications have not been established [53,54].

### 4.8. Skin Microbiome Modulation

Magnesium may influence the skin microbiome indirectly through interactions with the epidermal barrier, immune system, and local physicochemical environment. The skin microbiome contributes to barrier defense and immune homeostasis, and dysbiosis—particularly expansion of *Staphylococcus aureus*—is associated with inflammatory skin diseases such as atopic dermatitis (AD) [71]. 

Evidence specifically linking magnesium to microbiome modulation is still limited and largely indirect. Experimental work has shown that calcium and magnesium concentrations can influence bacterial growth on the skin. In a murine model, depletion of Ca^2+^ and Mg^2+^ from the washing environment suppressed *Staphylococcus aureus* growth both in vitro and on tape-stripped skin, suggesting that divalent cations can affect the cutaneous microbial environment. However, this study does not demonstrate that magnesium supplementation improves the skin microbiome; rather, it indicates that Mg^2+^ availability can influence microbial colonization [72]. 

More direct evidence comes from studies of Dead Sea climatotherapy, where magnesium is one of the predominant minerals. In patients with AD, Dead Sea climatotherapy was associated with partial normalization of microbial dysbiosis and significant changes in the relative abundance of several bacterial taxa, including *Staphylococcus epidermidis*, *Streptococcus mitis*, and *Micrococcus luteus*. Importantly, however, climatotherapy involves multiple environmental exposures—including mineral-rich water, sunlight, humidity, and climate—so these microbiome changes cannot be attributed specifically to magnesium [73]. 

Magnesium may also affect the microbiome indirectly by improving epidermal barrier integrity and modulating cutaneous inflammation. A controlled clinical study found that bathing in magnesium-rich Dead Sea salt improved skin hydration and barrier function and reduced redness in subjects with atopic dry skin. Since barrier disruption and inflammation can alter microbial colonization, these effects could theoretically contribute to a more stable microbial ecosystem. Nevertheless, the study did not directly measure the microbiome and the formulation contains several minerals, making it difficult to attribute the effects specifically to magnesium and calcium [33]. 

The current dermatological literature therefore supports a biologically plausible but not yet established role for magnesium in skin microbiome modulation. Indeed, magnesium may influence microbial ecology through effects on barrier function, immune signaling, and the ionic environment rather than acting as a proven microbiome-modulating agent. 

Table 2 provides an overview of the physiological roles of magnesium in maintaining skin health.

## 5. Magnesium in Skin Aging and Specific Dermatologic Disorders

### 5.1. Skin Aging

Maintaining adequate magnesium levels may contribute to healthy skin aging by modulating oxidative stress, inflammation, collagen metabolism, and cellular repair [3]. These functions provide a biological rationale for a potential role of magnesium in the aging skin process. However, clinical evidence for magnesium as an anti-aging intervention remains limited. Skin aging is strongly associated with oxidative stress, chronic low-grade inflammation, mitochondrial dysfunction, and progressive extracellular-matrix degradation, particularly loss and fragmentation of collagen and elastin [78]. Magnesium inadequacy has been associated with increased oxidative stress and inflammatory signaling during aging, suggesting that insufficient magnesium status could potentially contribute to processes involved in tissue aging [3,77]. However, this relationship remains largely mechanistic and does not establish that magnesium deficiency directly causes skin aging. Experimental evidence suggests that magnesium may have protective effects on skin cells. In vitro studies have shown that magnesium chloride can reduce UVB-associated reactive oxygen species, while magnesium has also been shown to influence fibroblast migration and collagen-related processes [20]. These findings are relevant because UV-induced oxidative stress is a major contributor to photoaging. There is also preliminary evidence for a direct effect on collagen metabolism. Experimental research involving magnesium lithospermate B demonstrated increased expression of collagen-related genes, including COL1A1 and COL3A1, in models of skin aging [79]. However, magnesium lithospermate B is a specific magnesium-containing compound derived from *Salvia miltiorrhiza*, and these findings should not be interpreted as evidence that conventional oral magnesium supplementation has anti-aging effects in humans. In a small, single-blind, placebo-controlled study of 20 healthy volunteers, a liposomal formulation containing magnesium ascorbyl phosphate, α-lipoic acid, and kinetin significantly improved skin hydration after 4 weeks, although TEWL and viscoelasticity were unchanged [80]. However, because magnesium was included in a multi-ingredient formulation, its specific contribution to the observed effects cannot be established.

Overall, magnesium may contribute to maintaining skin integrity and potentially modulate pathways involved in oxidative stress, inflammation, collagen synthesis, and cellular repair. Nevertheless, clinical evidence specifically demonstrating that magnesium supplementation prevents or reverses skin aging is currently limited. Thus, magnesium should be regarded as a potential supportive factor in skin health rather than an established anti-aging treatment. Further controlled clinical studies are needed to determine whether correcting magnesium deficiency or using topical magnesium formulations can produce measurable improvements in skin aging.

### 5.2. Atopic Dermatitis

Magnesium may play a role in AD by contributing to epidermal barrier homeostasis through calcium-dependent signaling involved in keratinocyte differentiation and barrier repair, as well as modulating inflammation, oxidative stress, and cutaneous immune regulation. However, the clinical evidence linking magnesium status to AD remains limited and heterogeneous, and magnesium should not currently be considered an established treatment for AD. Experimental studies have shown that magnesium salts can accelerate recovery of the epidermal permeability barrier after disruption, with the Ca^2+^/Mg^2+^ ratio appearing to influence the magnitude of barrier recovery [70]. This is potentially relevant to AD, in which impaired barrier function and increased TEWL are characteristic features. 

One of the most directly relevant clinical studies evaluated magnesium-rich Dead Sea salt bathing in patients with atopic dry skin. Bathing in a 5% Dead Sea salt solution significantly improved stratum corneum hydration and reduced skin roughness and redness. In participants with impaired baseline barrier function, TEWL was also reduced, suggesting improvement in epidermal barrier integrity [33]. Because Dead Sea salts contain multiple minerals and bathing itself may influence the skin, these effects cannot be attributed exclusively to magnesium. 

Magnesium may additionally influence cutaneous inflammation and immune responses. Experimental studies indicate that magnesium availability can modulate inflammatory cytokine production, oxidative stress, and immune-cell activation [15,16,17,18]. Of particular relevance to AD, magnesium ions have been shown experimentally to inhibit the antigen-presenting function of human epidermal Langerhans cells, reducing expression of HLA-DR and costimulatory molecules and decreasing TNF-α production [58]. These findings suggest a potential role for magnesium in limiting excessive cutaneous immune activation, although their clinical significance in AD remains uncertain. 

The relationship between magnesium and oxidative stress may also be relevant to AD. Magnesium deficiency has been associated with increased inflammatory and oxidative responses, while experimental studies demonstrate that magnesium can influence antioxidant defenses and cellular responses to ROS [15,16,17,18]. Since oxidative stress contributes to epidermal barrier dysfunction and inflammatory signaling in AD, adequate magnesium availability could theoretically support cutaneous homeostasis; however, direct clinical evidence demonstrating that magnesium supplementation improves oxidative stress or disease severity in AD is currently insufficient. 

Magnesium may also interact with the skin microbiome, another important component of AD pathophysiology. Dead Sea climatotherapy has been associated with changes in the skin microbial community in patients with AD, including alterations in *Staphylococcus* and other bacterial taxa [73]. Nevertheless, these effects cannot be attributed specifically to magnesium because climatotherapy involves simultaneous exposure to mineral-rich water, sunlight, and environmental factors. 

Overall, the available evidence suggests that magnesium may influence several pathways relevant to AD—including epidermal barrier repair, keratinocyte function, immune regulation, inflammation, oxidative stress, and potentially microbial homeostasis. However, most mechanistic evidence is experimental, while human evidence is limited and often involves magnesium-containing preparations rather than isolated magnesium. Therefore, further randomized controlled trials are needed to determine whether magnesium supplementation or topical magnesium has a clinically meaningful effect on AD severity.

### 5.3. Psoriasis

Magnesium may be relevant to psoriasis through its potential effects on keratinocyte proliferation, immune regulation, inflammation, and oxidative stress. Psoriasis is characterized by excessive keratinocyte proliferation and abnormal differentiation together with chronic activation of the IL-23/Th17 inflammatory axis. Because magnesium participates in cellular signaling, enzymatic reactions, and immune regulation, alterations in magnesium homeostasis have been proposed as a potential contributor to psoriatic inflammation [57]. 

Observational evidence suggests an association between magnesium status and psoriasis risk, although findings are not consistent. A recent analysis of 17,883 US adults found that increasing magnesium depletion scores were associated with a higher prevalence of psoriasis; participants with a depletion score ≥ 3 had approximately twice the odds of psoriasis compared with those with a score of 0 (OR 2.01, 95% CI 1.27–3.18) [81]. A large multicenter cohort study published in 2026 similarly reported that hypomagnesemia was associated with an increased risk of psoriasis (RR 1.17, 95% CI 1.10–1.25) [29]. These findings support an association but do not establish that magnesium deficiency causes psoriasis.

Case–control studies have reported inconsistent findings regarding serum magnesium in psoriasis. Basavaraj et al. found significantly higher serum magnesium concentrations in patients with both mild and severe psoriasis compared with controls [30]. In contrast, Kirmit et al., in a study of 87 patients with psoriasis and 60 healthy controls, found no significant difference in serum magnesium between groups, despite significant alterations in several oxidative-stress and inflammatory parameters, including increased ischemia-modified albumin, catalase, ferroxidase, copper, and CRP, together with reduced selenium, zinc, and albumin [82]. Such discrepancies may reflect differences in disease severity, diet, medications, renal function, and the limitations of serum magnesium as a measure of total-body magnesium status.

A potential mechanism involves the relationship between magnesium and inflammatory signaling. Magnesium deficiency has been associated with increased NF-κB activation and production of inflammatory mediators, including TNF-α and IL-6, while magnesium can modulate immune-cell activation and inflammatory responses. Because these pathways are central to psoriasis pathogenesis, magnesium status could potentially influence inflammatory activity [81]. However, evidence that magnesium supplementation specifically improves psoriasis through these mechanisms remains insufficient.

Magnesium may also be relevant to the oxidative-stress component of psoriasis. Psoriasis is associated with an imbalance between oxidant production and antioxidant defenses. In a clinical study, patients with psoriasis demonstrated alterations in oxidative-stress markers and inflammatory parameters, although serum magnesium itself did not differ significantly from controls [82]. Thus, magnesium may participate in the broader redox and inflammatory network without necessarily being a reliable standalone biomarker of disease activity.

Magnesium is essential for vitamin D metabolism and activation, as several enzymes involved in these processes are magnesium-dependent [74,75]. This interaction may be relevant to immune regulation, given vitamin D’s role in keratinocyte proliferation and maturation and its ability to modulate inflammation and pro-inflammatory cytokine production [75,83]. Magnesium deficiency may impair vitamin D metabolism and synthesis, whereas adequate magnesium status may support vitamin D availability [75]. Because lower serum vitamin D levels have been associated with psoriasis and greater disease severity, these findings suggest a potential indirect relationship between magnesium status, vitamin D metabolism, and psoriasis. 

Magnesium may also influence phospholipase A2 activity, which is elevated in psoriatic epidermis. Through competitive inhibition of calcium-dependent phospholipase A2, magnesium may attenuate cutaneous inflammation [84], providing another potential mechanism by which magnesium could contribute to the management of psoriasis.

Nevertheless, evidence for magnesium as a treatment for psoriasis remains limited. Magnesium-containing preparations have historically been used in dermatology, and magnesium-rich mineral therapies may have effects on inflammation and epidermal physiology. However, current clinical evidence consists mainly of observational studies, small trials, and studies in which magnesium is combined with other minerals or therapeutic interventions. Similarly, reviews of nutritional interventions in psoriasis consider the evidence for most dietary supplements to be inconsistent [85]. 

Overall, magnesium may influence psoriasis through interconnected effects on keratinocyte biology, immune regulation, inflammation, and oxidative stress. Current observational evidence suggests that magnesium depletion or hypomagnesemia may be associated with a higher risk of psoriasis, but the available data do not establish causality or support magnesium supplementation as a stand-alone psoriasis treatment. Further randomized controlled trials are needed to determine whether correcting magnesium deficiency can improve psoriasis severity or treatment response. 

### 5.4. Acne Vulgaris

Magnesium may have a role in acne vulgaris through its potential effects on inflammation, oxidative stress, keratinocyte biology, and microbial activity. However, the evidence specifically linking magnesium status to acne remains limited and inconsistent. A recent dermatological review concluded that available studies suggest a possible association between magnesium and acne severity, but emphasized that the clinical evidence is based mainly on small observational studies and requires confirmation in larger trials [57]. 

Several studies have investigated serum magnesium concentrations in patients with acne, with conflicting findings. A case–control study of 45 male patients found that serum magnesium was significantly lower in patients with severe acne compared with those with mild or moderate disease, although differences between the overall acne group and controls were not clearly established [86]. Similarly, a study of 60 patients reported significantly lower magnesium concentrations in severe compared with mild acne, while overall magnesium levels did not differ significantly between patients and controls [87]. Conversely, a 2026 case–control study involving 60 patients with acne and 60 healthy controls found no significant differences in serum magnesium between acne patients and controls or between different acne-severity groups, suggesting that circulating magnesium may not be a consistent biomarker of acne severity [88]. 

The potential biological relationship between magnesium and acne is plausible because magnesium participates in immune regulation and inflammatory signaling. As already discussed, magnesium deficiency has been associated with increased production of inflammatory mediators and oxidative stress, processes that are relevant to inflammatory acne lesions. In addition, magnesium may influence cellular responses involved in keratinocyte proliferation and differentiation, potentially affecting follicular epithelial homeostasis [19,53].

There is also preliminary evidence for a direct topical effect of magnesium. A pilot study evaluated magnesium microneedle patches in 19 patients with mild-to-moderate acne. Treatment was associated with significant improvement in acne lesion scores after seven days, and the authors proposed that magnesium could exert anti-inflammatory and antimicrobial effects, including activity against *Cutibacterium acnes* [89]. However, the small sample size, short follow-up, and absence of a robust control group substantially limit the strength of these findings. 

A randomized single-blind study also investigated an oral formulation containing magnesium, phosphate, omega-6, and omega-7 in patients with resistant acne. Although the supplemented group showed substantial clinical improvement, the formulation contained several active components, making it impossible to determine whether magnesium itself was responsible for the observed effect [90]. 

More recently, a large multicenter cohort study reported an association between abnormal serum magnesium levels and the risk of inflammatory skin diseases, including acne. This supports the hypothesis that magnesium dysregulation may be relevant to inflammatory skin disease, but observational data cannot establish whether magnesium abnormalities contribute causally to acne or are consequences of other metabolic or lifestyle factors [29]. 

Overall, magnesium may influence acne vulgaris through inflammatory, oxidative, epithelial, and potentially antimicrobial mechanisms, but current evidence remains preliminary. Importantly, the available studies do not establish magnesium deficiency as a cause of acne, nor do they provide sufficient evidence to recommend magnesium supplementation as a standard acne treatment. Larger randomized controlled trials using well-defined magnesium preparations are needed to clarify whether correcting magnesium deficiency or applying magnesium topically has meaningful therapeutic effects.

### 5.5. Rosacea

Rosacea is a chronic inflammatory disorder involving innate immune activation, neurovascular dysregulation, oxidative stress, and abnormal responses to environmental stimuli. Because magnesium participates in immune and vascular signaling and modulates cellular responses to oxidative stress, magnesium homeostasis could potentially influence pathways involved in rosacea pathogenesis. However, direct clinical evidence specifically linking magnesium to rosacea remains limited. The relationship between magnesium and vascular function is also of potential interest because persistent facial erythema and episodic flushing are characteristic features of rosacea. Magnesium can influence vascular smooth-muscle tone and endothelial function through interactions with calcium-dependent signaling and nitric-oxide pathways [63,64,65]. Nevertheless, evidence that magnesium supplementation specifically improves cutaneous vasodilation, flushing, or telangiectasia in rosacea is currently lacking. Thus, the vascular rationale remains mechanistic rather than clinically established.

A recent two-sample Mendelian randomization study specifically investigated genetically predicted serum levels of magnesium and several other micronutrients in relation to rosacea. Importantly, the study found no statistically significant causal association between genetically predicted serum magnesium levels and rosacea risk, whereas higher genetically predicted calcium levels were associated with increased rosacea risk and potassium levels with reduced risk [91]. These findings provide useful evidence against assuming a direct causal relationship between circulating magnesium levels and rosacea, although Mendelian randomization evaluates genetically determined exposure and does not exclude effects of acquired magnesium deficiency or topical magnesium. 

Evidence from reviews of micronutrients in rosacea is similarly inconclusive. A 2024 systematic review identified studies investigating several vitamins and minerals, including magnesium, but concluded that there are currently no established recommendations for micronutrient supplementation in rosacea [92]. Additional studies evaluating serum nutrient levels and clinical outcomes are required. 

Magnesium could also theoretically influence rosacea through oxidative stress and epidermal barrier function. Experimental studies in keratinocytes indicate that magnesium can reduce oxidative damage, including UVB-induced ROS generation [26], while magnesium-containing preparations have demonstrated barrier-supportive effects in other inflammatory skin conditions [29]. However, extrapolation of these findings to rosacea should be made cautiously because these effects have not been adequately tested in rosacea-specific clinical trials. 

Overall, magnesium has plausible anti-inflammatory, antioxidant, vascular, and barrier-related effects that could intersect with rosacea pathophysiology, but current evidence does not establish magnesium deficiency as a cause of rosacea or magnesium supplementation as an effective treatment. The most recent genetic evidence found no significant causal association between serum magnesium and rosacea risk, highlighting the need for well-designed clinical studies before magnesium can be considered a therapeutic intervention in rosacea [91]. 

### 5.6. Chronic Urticaria

Chronic urticaria (CU) is defined by the recurrent occurrence of wheals, angioedema, or both for more than six weeks. It can be classified as chronic spontaneous urticaria (CSU), in which symptoms occur without a consistent external trigger, or chronic inducible urticaria, in which symptoms are reproducibly triggered by physical or environmental stimuli. Mast-cell activation and the subsequent release of histamine and other inflammatory mediators are central to the pathophysiology of urticaria [76]. Magnesium is involved in numerous cellular processes, including calcium signaling, inflammation, oxidative stress, and cellular excitability, providing a theoretical basis for a possible role in mast-cell regulation. Although magnesium can influence inflammatory and histamine-related pathways in experimental and other allergic conditions [93,94,95,96], these findings cannot be directly extrapolated to chronic urticaria. 

Although magnesium is not currently recommended as a specific treatment for CSU in international evidence-based guidelines, which recommend second-generation H1-antihistamines followed, when necessary, by dose escalation, omalizumab, and ciclosporin for refractory cases [76], this absence of a recommendation should not be interpreted as definitive evidence of ineffectiveness. Rather, it reflects the limited clinical evidence currently available to support its therapeutic use.

The relationship between magnesium and CU remains an interesting but insufficiently investigated area. Magnesium has plausible immunological and cellular effects that could theoretically influence mast-cell activation, inflammatory signaling, and oxidative stress. However, current evidence does not demonstrate magnesium deficiency as a direct cause of CU or supplementation as an effective CSU treatment. Magnesium may be considered when a patient has a documented deficiency, but it should not currently be regarded as a specific treatment for CU. Further randomized controlled studies are needed to determine whether magnesium status or supplementation has any clinically meaningful effect on CSU.

### 5.7. Wound Healing

Experimental studies suggest that adequate extracellular magnesium is important for endothelial-cell proliferation and migration, processes that are essential for new blood-vessel formation during wound healing [63,64]. Magnesium may also influence inflammatory signaling and cellular responses involved in tissue regeneration [60]. Recent experimental research has provided additional evidence for a direct role of magnesium in cutaneous repair. In human keratinocyte models, MgCl_2_ increased expression of matrix metalloproteinase-7 (MMP-7) and enhanced cell migration through activation of the MEK/ERK pathway, suggesting a potential role for magnesium in the early stages of epithelial repair [20]. Other preclinical studies of magnesium-containing biomaterials, often incorporating additional active components, have reported enhanced fibroblast migration, neovascularization, basal-cell proliferation, collagen deposition, and modulation of inflammatory responses in experimental wound models [97,98]. Magnesium-containing hydrogels have also demonstrated antibacterial activity and accelerated healing in infected full-thickness skin wounds in mice [99]. These studies support the potential of magnesium-containing formulations, but they do not establish that magnesium alone is responsible for the observed effects.

Clinical evidence remains limited to a small number of trials [100,101] with relatively small sample sizes and focused on magnesium supplementation in patients with diabetic foot ulcers. Therefore, these findings should be interpreted cautiously and cannot yet be generalized to other types of wounds.

Overall, the available evidence suggests that magnesium may support wound healing through effects on cell migration and proliferation, angiogenesis, inflammation, collagen/extracellular-matrix formation, and potentially antimicrobial activity. However, much of the evidence remains preclinical, and clinical trials are still limited. Therefore, magnesium should currently be considered a potential supportive factor in wound healing rather than an established stand-alone treatment. Correcting a documented magnesium deficiency may be clinically appropriate, but further well-designed clinical trials are needed to establish the efficacy, optimal dose, formulation, and route of magnesium administration for wound management.

### 5.8. Diabetic Foot Ulcers

The potential role of magnesium diabetic foot ulcer (DFU) healing may be explained through effects on glucose metabolism, inflammation, oxidative stress, and tissue repair. Hypomagnesemia has been associated with diabetic complications, including neuropathy and abnormal platelet activity [35,36,41,102], which may contribute to the development and impaired healing of DFUs. Clinical evidence is limited but encouraging. 

In a randomized, double-blind, placebo-controlled trial involving 70 patients with grade 3 DFUs, oral supplementation with 250 mg/day magnesium oxide for 12 weeks significantly reduced ulcer length, width, and depth compared with placebo. Magnesium supplementation also improved glycemic parameters, reduced high-sensitivity CRP, and increased total antioxidant capacity [101]. A subsequent randomized trial of 57 patients found that oral magnesium oxide (250 mg/day) combined with vitamin E (400 IU/day) for 12 weeks significantly reduced ulcer length, width, and depth compared with placebo, while also improving glycemic control and markers of oxidative stress and inflammation [100]. However, the independent contribution of magnesium remains unclear due to its combined administration with vitamin E.

These findings suggest that magnesium may support DFU healing partly by improving metabolic control, reducing inflammation and oxidative stress, and potentially facilitating tissue repair. However, the available trials are relatively small, and the evidence is not sufficient to establish magnesium as a standard treatment for DFUs. A Cochrane review identified magnesium among several nutritional interventions studied for diabetic foot ulcers but emphasized the limited evidence base for nutritional supplementation overall [103].

Overall, magnesium supplementation may be beneficial, particularly in patients with inadequate magnesium status, but it should be considered an adjunct to standard DFU management rather than a replacement for evidence-based wound care, off-loading, infection management, vascular assessment, and glycemic control.

### 5.9. Pressure Ulcers

As discussed previously and although much of the evidence remains preclinical, magnesium is involved in cellular proliferation, protein synthesis, inflammation, angiogenesis, and tissue repair, all of which are relevant to wound healing. Indeed, magnesium may influence keratinocyte proliferation, fibroblast migration, collagen synthesis, and inflammatory responses [53]. Despite this biological rationale, direct clinical evidence for magnesium supplementation in pressure ulcers is very limited. Nutritional interventions are recognized as an important component of pressure-injury management, particularly in patients who are malnourished or nutritionally deficient. Current pressure-ulcer guidelines emphasize adequate energy, protein, and micronutrient intake, but they do not recommend magnesium supplementation specifically as a treatment for pressure ulcers [104]. 

A recent Cochrane review found that the evidence concerning calcium, magnesium, and vitamin D supplementation for pressure-ulcer prevention is very uncertain, with only limited studies directly evaluating these nutrients. Overall, the evidence is insufficient to determine whether magnesium supplementation prevents or accelerates pressure-ulcer healing [105]. As mentioned above, indirect evidence from other chronic wounds, in particular for DFU, is somewhat more encouraging [100,101]. However, these findings cannot be directly extrapolated to pressure ulcers.

Overall, magnesium may contribute to normal wound-healing processes, particularly when magnesium status is inadequate, but there is currently insufficient evidence to support magnesium supplementation as a specific treatment for pressure ulcers. Further randomized controlled trials are needed to determine whether magnesium status or supplementation has a clinically meaningful effect on pressure-ulcer prevention or healing.

### 5.10. Hair Disorders

Because hair follicles are highly metabolically active structures, adequate nutrient availability, including magnesium, is important for normal hair growth and follicular function [106]. However, the specific role of magnesium in hair disorders remains poorly established. Studies investigating alopecia areata (AA) have produced inconsistent results. Some studies found no significant difference in serum magnesium between patients with AA and healthy controls [107], while a meta-analysis of 10 studies involving 764 subjects also found no significant difference in serum magnesium levels between AA patients and controls [108]. Evidence for magnesium supplementation as a treatment for hair loss is also limited. A clinical study of nutritional supplements containing magnesium, together with zinc, biotin, iron, vitamins and amino acids, reported improvements in hair loss and hair density in patients with telogen effluvium; however, because magnesium was administered as part of a multi-ingredient formulation, its individual contribution cannot be determined [109]. Magnesium may nevertheless have a biological role in the hair follicle. Experimental research has demonstrated that magnesium ions can penetrate the skin, with hair follicles contributing significantly to this penetration, suggesting that follicles may represent an important pathway for topical magnesium delivery [110].

Overall, current evidence does not support magnesium deficiency as an established cause of alopecia or magnesium supplementation as a proven treatment for hair disorders. Evidence is particularly insufficient for androgenetic alopecia, telogen effluvium, and alopecia areata. Magnesium supplementation may be appropriate when a clinically documented deficiency exists, but routine supplementation specifically for hair loss cannot currently be recommended on the basis of available evidence. 

### 5.11. Nail Disorders

The normal nail plate contains magnesium as part of its mineral composition, along with calcium, iron, zinc, sodium, and copper [111]. Despite this biological role, evidence directly linking magnesium status to nail disorders is limited. Nutritional deficiencies can affect nail growth and morphology, but the best-established nutritional associations with nail abnormalities involve iron and zinc rather than magnesium [112,113]. In particular, iron deficiency and zinc deficiency have been associated with brittle or dystrophic nails, whereas a specific clinical phenotype caused by magnesium deficiency has not been clearly established. Some evidence suggests that magnesium may have a supportive role in nail growth. A combination of magnesium biotinate and arginine silicate has been reported to increase nail growth, but the available studies were small and, in some cases, lacked a control group; therefore, the specific contribution of magnesium cannot be determined [112]. Similarly, recent reviews of nutritional and vitamin-based treatments for nail disorders emphasize that evidence for supplementation remains limited and that treatment should focus primarily on identifying and correcting the underlying cause of nail abnormalities [112,114]. 

Overall, magnesium is biologically relevant to nail structure and growth, but there is currently insufficient evidence to establish magnesium deficiency as a cause of nail disorders or magnesium supplementation as an effective treatment for brittle nails, onycholysis, nail dystrophy, or other common onychopathies. Magnesium supplementation may be appropriate when a documented deficiency is present, but routine supplementation specifically for nail health cannot currently be recommended based on the available evidence.

### 5.12. Autoimmune Skin Diseases

As previously discussed, low magnesium status may promote inflammation by enhancing cytokine production and activating immune and endothelial cells [15,16,17,18], providing a potential biological link between magnesium homeostasis and immune-mediated skin disease. Furthermore, hypomagnesemia was found associated with inflammatory skin disorders, including psoriasis and atopic dermatitis, in a recent multicenter cohort study. However, the observational design of these results does not establish causality or demonstrate that magnesium supplementation prevents or treats these diseases [29]. Studies of psoriasis, an example of an autoimmune disease, have also reported alterations in serum magnesium concentrations, but results have been inconsistent, and the clinical significance of these findings remains uncertain [82,83]. The most direct evidence concerning an autoimmune skin disorder comes from experimental research in systemic lupus erythematosus (SLE). In a murine lupus model, a high-magnesium diet reduced skin lesions and histological disease severity, decreased pathogenic anti-dsDNA autoantibodies, and increased regulatory T cells [115]. These findings suggest that magnesium may influence immune tolerance and autoimmune activity; however, they cannot yet be extrapolated to patients with human SLE.

Ichthyosis, not an autoimmune but an inherited disorder affecting skin formation and shedding, may provide additional insight into the relationship between epidermal lipid metabolism, ionic homeostasis, and hyperplasia. Group IIF secreted phospholipase A_2_ contributes to epidermal barrier maintenance and keratinocyte differentiation, while its dysregulation may promote psoriasis-like hyperplasia. The calcium/magnesium ratio may also influence epidermal homeostasis, although its specific role in ichthyosis requires further investigation [116,117].

Vitiligo is an acquired depigmenting autoimmune disorder characterized by the loss of melanocytes. Magnesium may contribute to melanocyte function and antioxidant defense, potentially influencing mechanisms involved in vitiligo pathogenesis. However, evidence linking magnesium status to vitiligo remains limited, and further research is needed to clarify its clinical significance [118].

Overall, magnesium appears to have immunomodulatory and anti-inflammatory properties that may be relevant to autoimmune and inflammatory skin diseases, but clinical evidence remains limited. At present, there is insufficient evidence to recommend magnesium supplementation as a specific treatment for psoriasis, lupus, or other autoimmune skin disorders. Correction of a documented magnesium deficiency may nevertheless be appropriate as part of general nutritional management, while further clinical trials are needed to determine whether magnesium supplementation has therapeutic benefits in these conditions. 

Table 3 examines magnesium’s potential contribution to cutaneous aging and a range of skin diseases.

## 6. Available Magnesium-Containing Formulations

### 6.1. Oral Magnesium

**Figure 2:**
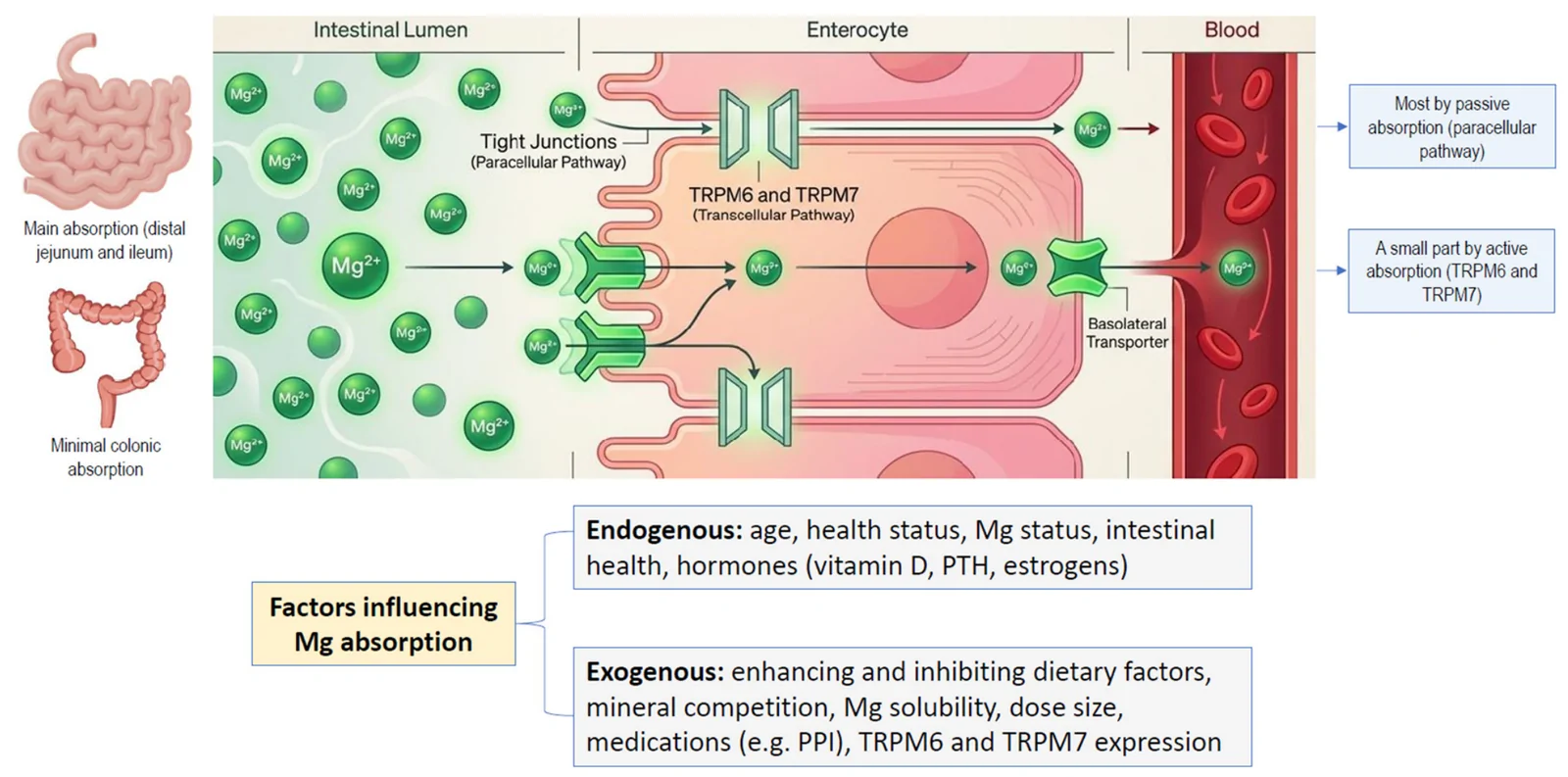
Magnesium absorption and factors affecting its bioavailability.

Different magnesium salts exhibit distinct physicochemical and pharmacokinetic properties. Several soluble organic salts, including citrate, lactate, aspartate, and glycinate, generally show greater bioavailability than poorly soluble magnesium oxide; however, magnesium chloride is highly soluble and should not be grouped with poorly absorbed magnesium oxide [119,120,121,122]. Bioavailability depends not only on salt chemistry but also on dose, gastric and intestinal conditions, baseline magnesium status, and formulation [122,123,124] (Figure 2). Magnesium is absorbed mainly in the small intestine and, to a lesser extent, in the colon through passive paracellular and active transcellular mechanisms. Amino acid-chelated forms such as magnesium glycinate have also been proposed to involve peptide-transport pathways, although comparative human mechanistic evidence remains limited [123]. Table 4 summarizes the main characteristics of commonly used oral magnesium salts. 

From a safety perspective, high supplemental doses may cause gastrointestinal adverse effects, particularly diarrhea, making gradual dose titration advisable. Renal function should also be considered before supplementation, as magnesium is primarily eliminated by the kidneys and supplementation requires caution in patients with renal impairment. Novel magnesium delivery systems, including microencapsulation and liposomal formulations, may improve bioavailability, prolong magnesium release, and enhance gastrointestinal tolerability [121,125,126]. However, their clinical advantages over conventional magnesium supplements remain to be established through well-designed studies.

**Table 4:** Main characteristics of the available oral magnesium salts.

Oral Mg Formulation	Approx. Elemental Mg *	Relative Bioavailability	Typical Adult Supplemental Dose **	Main Advantages/Considerations	Safety & Tolerability	Ref.
Magnesium citrate	~11–16%	Generally good; better than oxide in several human studies	100–300 mg elemental mg/day	Soluble formulation; widely available; useful for supplementation	Diarrhea and abdominal discomfort at higher doses	[2,121,127,128,129]
Magnesium chloride	~12% for hexahydrate; ~25.5% anhydrous	Generally good; comparable to lactate and aspartate in a human study	100–300 mg/day	Highly soluble; available in oral liquid and solid preparations	Diarrhea and gastrointestinal discomfort possible	[2,121,127,129]
Magnesium lactate	~10–12%, depending on hydration	Good in comparative human studies	100–300 mg/day	Relatively well studied; suitable for oral replacement	Gastrointestinal effects possible	[2,121,127,129]
Magnesium aspartate	Product-dependent; approximately 8–10% for common forms	Good in comparative human studies	100–300 mg/day	Human evidence supports absorption comparable to chloride and lactate	Gastrointestinal effects possible; consider the full formulation and clinical context	[2,121,127]
Magnesium gluconate	~5–6%	Moderate to good; formulation-dependent	100–300 mg/day	Used in oral magnesium replacement; lower elemental content per unit mass	Diarrhea and gastrointestinal discomfort possible	[121,129]
Magnesium glycinate/bisglycinate	Approximately 10–14%, depending on composition	Evidence is limited for claims of superiority over other well-absorbed forms	100–300 mg/day	Amino-acid chelate; often marketed for gastrointestinal tolerability, but robust comparative evidence is limited	Generally tolerated; comparative clinical data remain limited	[2,128]
Magnesium malate	Approximately 15% for anhydrous magnesium malate; varies by formulation	Insufficient comparative human evidence for a confident ranking	100–300 mg/day	Organic salt; marketed for supplementation, but specific advantages are not firmly established	Gastrointestinal effects possible; limited formulation-specific data	[128]
Magnesium oxide	~60.3%	Relatively low in several human comparative studies	200–350 mg/day, depending on indication and clinical guidance	High elemental magnesium content; inexpensive; lower solubility	Diarrhea and abdominal discomfort; excessive intake can cause toxicity	[2,121,127,128,129]
Magnesium hydroxide	~41.7%	Not generally compared as a routine supplement; used mainly as an antacid or laxative	Usually used according to the medicinal product’s therapeutic instructions	Useful for acid neutralization and constipation rather than routine supplementation	Laxative effects are expected; excessive use can cause hypermagnesemia, particularly with renal impairment	[129,130]
Magnesium carbonate	~28.8% for anhydrous MgCO_3_; commercial basic carbonates vary	Variable; depends on compound and preparation	Product- and indication-dependent	Used in some supplements and antacid preparations	Gastrointestinal effects possible; excessive magnesium can cause toxicity	[129,130]
Magnesium sulfate	~10% for the heptahydrate; ~20% anhydrous	Relatively low or variable orally; commonly used clinically by other routes	Not generally preferred for routine oral supplementation	Oral preparations have a prominent osmotic laxative effect; distinguish oral use from medical intravenous administration [1,2,6]	Diarrhea and dehydration; excessive exposure can cause hypermagnesemia	[129,130]

* Elemental magnesium content: Approximate percentages refer to the magnesium fraction by mass of the specified chemical compound. Hydration state, chelate composition, and commercial formulation can substantially alter the actual percentage. The product label’s stated elemental magnesium content should take precedence. ** Supplemental dose: These ranges are illustrative adult amounts, not universal recommendations or treatment prescriptions. Dose selection depends on dietary intake, indication, renal function, concomitant medication, and local guidance. The amount of elemental magnesium—not the total mass of the salt—should be used when calculating the dose. Abbreviations: GI: gastrointestinal; Mg: magnesium; Ref.: references.

### 6.2. Topical Magnesium 

Topical magnesium is available primarily as magnesium chloride (MgCl_2_) and magnesium sulfate (MgSO_4_) in aqueous solutions, sprays, creams, gels, and bath preparations; magnesium-containing hydrogels and wound dressings are also being investigated. These two most commonly used topical salts (MgCl_2_ and MgSO_4_) may have potential applications in skin-barrier function, inflammation, wound healing, and photoprotection. However, most clinical evidence remains limited and formulations are heterogeneous [57]. Magnesium chloride has been investigated in experimental skin models, where topical application enhanced keratinocyte migration through MMP-7 and MEK/ERK signaling, suggesting a potential role in early wound repair [20]. Magnesium-containing creams have also shown promising effects in preclinical burn models, including increased fibroblast and collagen formation, angiogenesis, and reduced inflammatory markers [23]. Topical magnesium may also influence skin-barrier recovery. Experimental studies have reported accelerated barrier repair following application of magnesium salts, particularly magnesium chloride, although these findings are primarily preclinical [70]. 

Importantly, systemic absorption through intact skin remains uncertain. A recent 2026 ex vivo study found negligible magnesium permeation through intact skin from magnesium sulfate solution, whereas disruption of the stratum corneum markedly increased penetration [131]. Thus, topical magnesium should currently be considered primarily for potential local dermatological effects rather than as a reliable method of correcting systemic magnesium deficiency.

## 7. Current Research Gaps and Future Directions

Interest in magnesium as a potential modulator of skin health has increased, with experimental and preliminary clinical evidence suggesting possible roles in skin-barrier function, inflammation, wound healing, oxidative stress, photoprotection, and immune regulation. However, the current evidence remains heterogeneous and insufficient to establish magnesium as a standard treatment for most dermatologic disorders. In fact, available studies are frequently limited by small sample sizes, heterogeneous magnesium formulations and doses, variable treatment durations, and inconsistent clinical outcomes. 

A major research gap is the lack of well-designed, adequately powered randomized controlled trials. Existing clinical studies have mainly evaluated specific populations, such as patients with DFU or AD, and results cannot necessarily be extrapolated to other dermatologic conditions. For example, magnesium supplementation has shown encouraging effects on wound-healing parameters in DFU [100,101], but these findings require confirmation in larger and more diverse patient populations. Similarly, recent observational data suggest an association between hypomagnesemia and psoriasis [81], but these studies cannot determine whether low magnesium is a cause, consequence, or marker of disease.

Another important gap concerns magnesium status assessment. Serum magnesium is widely available but may not accurately reflect total-body or intracellular magnesium status. Future dermatological studies should therefore consider standardized approaches combining serum magnesium with dietary intake, renal function, urinary magnesium excretion, and, where feasible, more sensitive intracellular or functional biomarkers. This would help identify whether particular dermatologic phenotypes are associated with genuine magnesium deficiency and whether patients with low magnesium status respond preferentially to supplementation.

The optimal formulation, dose, route, and duration of magnesium treatment also remain unclear. Different salts have different physicochemical properties and bioavailability, while topical preparations—including magnesium chloride, magnesium sulfate, creams, gels, and magnesium-containing wound dressings—may have substantially different pharmacokinetic profiles. Recent experimental evidence indicates that magnesium permeation through intact skin may be negligible [131], highlighting the need for rigorous studies of topical absorption and tissue distribution before systemic effects are attributed to transdermal preparations.

Mechanistic research should further clarify how magnesium influences keratinocyte differentiation, fibroblast migration, collagen and extracellular-matrix remodeling, inflammatory signaling, oxidative stress, and calcium-dependent pathways. For example, experimental studies demonstrate that MgCl_2_ can promote keratinocyte migration through the MMP-7/MEK/ERK pathway, supporting a potential role during early wound repair [20]. Future translational studies should determine whether these cellular effects are reproduced in human skin and whether they translate into clinically meaningful improvements.

Finally, future research should prioritize disease-specific clinical trials in psoriasis, atopic dermatitis, acne, chronic wounds, pressure ulcers, diabetic foot ulcers, hair and nail disorders, and photoaging. Trials should use standardized formulations, validated disease-specific endpoints, objective biomarkers of magnesium status, and long-term safety monitoring. Particular attention should be given to patients with conditions associated with altered magnesium metabolism, including diabetes and chronic kidney disease.

Overall, magnesium represents a promising but still investigational adjunct in dermatology. The next phase of research should move from mechanistic and small observational studies toward standardized, adequately powered randomized trials that establish which patients may benefit, which formulations and doses are most effective, and whether correction of magnesium deficiency itself improves dermatologic outcomes. 

## 8. Limitations

This narrative review has several limitations. First, the absence of a systematic search strategy and predefined inclusion and exclusion criteria may have introduced selection bias. Second, the available evidence is heterogeneous in terms of study design, populations, magnesium formulations, routes of administration, and clinical outcomes, limiting comparability across studies. Furthermore, much of the current evidence derives from preclinical studies or small clinical populations, restricting the generalizability of the findings. The limited clinical data on topical magnesium, particularly regarding its cutaneous absorption, optimal dosage, and long-term safety, further constrain conclusions about its therapeutic potential. Therefore, the findings should be interpreted cautiously, and further well-designed clinical trials are needed to establish the efficacy and safety of magnesium-based interventions in dermatological conditions.

## 9. Conclusions

Magnesium participates in skin-barrier function, cellular proliferation, inflammatory and redox signaling, and wound-healing processes, suggesting potential relevance across several dermatologic disorders. It should therefore be regarded as a biologically plausible modulator of pathways relevant to dermatologic disease rather than an established therapeutic target. However, current clinical evidence remains limited and heterogeneous, with many studies being preclinical or based on small populations. Topical magnesium may have local benefits, but its transdermal absorption remains uncertain. Overall, magnesium should currently be considered a potential adjunct rather than an established dermatologic treatment, and well-designed clinical trials are needed to define its efficacy, optimal formulations and doses, and long-term safety.

## Figures and Tables

**Table 1:** Methods for assessing magnesium status.

Assessment Method	What It Measures	Main Advantages	Main Limitations/Interpretation	Key Ref.
Serum/plasma total Mg	Total circulating Mg (free + protein/anion-bound)	Widely available, inexpensive, routinely used; useful for detecting clinically significant hypomagnesemia	Poor marker of total-body Mg because <2% of body Mg is extracellular; may remain normal despite intracellular depletion	Witkowski et al. [49]
Ionized magnesium (iMg)	Biologically active, free Mg fraction	May better reflect physiologically active Mg than total serum Mg	Limited availability; affected by pH and sample handling; requires standardized measurement	Rayana et al. IFCC guidelines [50]
RBC magnesium	Intracellular Mg concentration in RBCs	May provide information about intracellular Mg; responds to dietary manipulation	Reference ranges and clinical interpretation are not standardized; RBC Mg does not necessarily reflect Mg status in other tissues	Witkowski et al. [49]
Urinary magnesium excretion	Renal Mg loss, usually measured in a 24-h urine collection	Useful for assessing renal Mg handling and response to changes in Mg intake	Influenced by dietary intake, renal function, medications and hormonal factors; not a direct measure of total-body Mg	Witkowski et al. [49]
Magnesium loading/retention test	Percentage of administered Mg retained after a Mg load, based on urinary excretion	Functional test that may detect Mg depletion despite a normal serum Mg	More complex and invasive; protocols are not standardized; unsuitable or difficult to interpret in renal dysfunction	Arnaud et al.; Witkowski et al. [49,51]
Leukocyte/platelet magnesium	Intracellular Mg in circulating cells	Potentially reflects intracellular status more directly than serum Mg	Mainly research-based; technically demanding and not routinely available	Rondón & Marín [44]
Muscle magnesium	Mg concentration in skeletal muscle	May better represent intracellular/tissue Mg	Requires muscle biopsy; therefore, unsuitable for routine clinical use	Elin; Arnaud et al. [51,52]
Bone magnesium	Mg stored in bone	Represents a major body Mg compartment	Requires invasive sampling and is not suitable for routine assessment	Arnaud et al. [51]
Hair/nail magnesium	Mg incorporated into keratinized tissues	Non-invasive and potentially useful for population/research studies	Strongly influenced by external contamination, growth and other factors; clinical validity remains uncertain	Rondón & Marín [44]
Dietary magnesium intake	Estimated Mg intake from food ± supplements	Simple and useful for nutritional assessment and epidemiology	Estimates intake rather than body Mg status; absorption and renal losses are not accounted for	Witkowski et al. [49]

Abbreviations: IFCC: International Federation of Clinical Chemistry and Laboratory Medicine; iMg: ionized magnesium; Mg: magnesium; RBC: red blood cells; ref.: references.

**Table 2:** Physiological functions of magnesium relevant to skin health.

Physiological Function	Role of Magnesium	Relevance to Skin Health	Ref.
Cellular energy metabolism	Acts as a cofactor for ATP-dependent reactions and numerous metabolic enzymes	Supports the high metabolic demands of keratinocytes, fibroblasts, and other skin cells	[1,2,3]
DNA and protein synthesis	Required for nucleic-acid synthesis, ribosomal function, and protein production	Supports cell proliferation, tissue renewal, and synthesis of structural proteins	[3,5]
Keratinocyte proliferation and differentiation	Modulates cellular signaling and proliferation pathways	Contributes to epidermal renewal and maintenance of skin integrity	[15,21]
Skin-barrier function	Influences epidermal differentiation and cellular homeostasis	May support barrier integrity and recovery following barrier disruption	[23,56]
Fibroblast activity	Influences fibroblast migration and proliferation	Relevant to wound repair and extracellular-matrix formation	[22]
Collagen and extracellular-matrix metabolism	Participates in enzymatic and signaling pathways involved in matrix remodeling	May contribute to dermal structure, wound healing, and maintenance of skin elasticity	[55,56]
Wound healing	Modulates keratinocyte migration, fibroblast activity, angiogenesis, and inflammatory responses	Supports the different phases of tissue repair	[15,21,22,23]
Inflammatory regulation	Influences cytokine production and immune-cell signaling	May help regulate cutaneous inflammatory responses	[15,16,17,18,27]
Oxidative-stress regulation	Contributes indirectly to antioxidant defenses and cellular redox homeostasis	May protect skin cells from oxidative damage associated with environmental stress and aging	[15,59,60]
Vitamin D metabolism	Required for enzymes involved in vitamin D synthesis and activation	May indirectly influence immune regulation and epidermal homeostasis	[74,75]
Calcium homeostasis and signaling	Interacts with calcium-dependent signaling pathways and regulates intracellular Mg^2+^/Ca^2+^ balance	Important for keratinocyte differentiation, barrier function, and inflammatory signaling	[21,53,62,69,70]
Mast-cell and immune regulation	Modulates cellular signaling involved in immune and inflammatory responses	Potential relevance to inflammatory and immune-mediated skin diseases	[16,76]
Angiogenesis	Influences endothelial-cell proliferation and migration	Supports vascularization during wound healing and tissue regeneration	[22,66]
Cellular senescence and aging	Magnesium status may influence oxidative stress, inflammation, and cellular metabolic pathways associated with aging	Potential relevance to intrinsic and extrinsic skin aging	[3,15,20,59,60,77,78,79,80]

Abbreviations: ATP: adenosine triphosphate; Ca^2+^: calcium; DNA: deoxyribonucleic acid; Mg^2+^: magnesium; Ref.: references.

**Table 3:** Summary of the potential role of magnesium in skin aging and specific dermatologic disorders.

Skin Condition	Potential Mechanisms of Magnesium	Key Evidence	Overall Evidence and Clinical Implications
Skin aging	Antioxidant and anti-inflammatory effects; modulation of collagen metabolism, fibroblast migration, and cellular repair; potential protection against UV-induced oxidative stress.	In vitro studies suggest protection against UVB-induced oxidative stress and effects on collagen-related pathways. A small study of a multi-ingredient topical formulation reported improved skin hydration, but magnesium’s independent contribution was unclear [3,20,77,78,79,80].	Limited clinical evidence. Magnesium may support skin integrity, but its efficacy as an anti-aging intervention has not been established.
Atopic dermatitis	Support of epidermal barrier repair and keratinocyte function; modulation of inflammatory cytokines, oxidative stress, and cutaneous immune responses; possible effects on the skin microbiome.	Magnesium-rich Dead Sea salt bathing improved hydration, roughness, and redness, with reduced TEWL in some participants. Experimental studies suggest immunomodulatory effects [33,58,70,73].	Limited and indirect evidence. Benefits cannot be attributed specifically to magnesium. Randomized controlled trials are needed to establish efficacy.
Psoriasis	Modulation of keratinocyte proliferation and differentiation, immune activation, inflammatory signaling, oxidative stress, and vitamin D metabolism; possible inhibition of calcium-dependent phospholipase A2.	Observational studies suggest an association between magnesium depletion or hypomagnesemia and psoriasis risk, but serum magnesium findings are inconsistent. A large cohort reported an association between hypomagnesemia and psoriasis [29,30,81,82,83,84].	Limited and inconsistent evidence. Association does not establish causality, and magnesium supplementation is not an established psoriasis treatment.
Acne vulgaris	Potential anti-inflammatory and antioxidant effects; modulation of keratinocyte biology and possible antimicrobial activity against *Cutibacterium acnes*.	Case–control studies report inconsistent serum magnesium findings. A small pilot study of magnesium microneedle patches showed improvement in acne lesion scores, while an oral multi-ingredient formulation also reported clinical improvement [86,87,88,89].	Preliminary evidence. Small samples, short follow-up, and combination formulations prevent firm conclusions regarding magnesium’s independent effects.
Rosacea	Potential modulation of vascular tone, endothelial function, inflammation, oxidative stress, and epidermal barrier function.	A two-sample Mendelian randomization study found no significant causal association between genetically predicted serum magnesium levels and rosacea risk. Reviews have not established a role for micronutrient supplementation [91,92].	Insufficient clinical evidence. Neither magnesium deficiency as a cause nor supplementation as an effective treatment has been established.
Chronic urticaria	Theoretical modulation of calcium signaling, mast-cell activation, histamine-related pathways, inflammation, and oxidative stress.	Evidence is primarily mechanistic or extrapolated from other allergic and inflammatory conditions. Magnesium is not a recommended specific treatment in current urticaria guidelines [76,93,94,95,96].	Insufficient evidence. Magnesium supplementation cannot currently be recommended as a specific treatment for chronic spontaneous urticaria.
Wound healing	Promotion of endothelial-cell migration and angiogenesis, keratinocyte proliferation and migration, fibroblast activity, extracellular-matrix formation, and inflammatory regulation; potential antimicrobial effects.	Preclinical studies report enhanced cellular migration, neovascularization, collagen deposition, and wound repair with magnesium or magnesium-containing biomaterials. Clinical studies remain limited [20,63,64,97,98,99,100,101]	Promising preclinical evidence but limited clinical validation. Magnesium may be a supportive factor rather than an established stand-alone wound treatment.
Diabetic foot ulcers (DFUs)	Potential improvement in glycemic control, inflammation, oxidative stress, and tissue repair.	A randomized controlled trial involving 70 patients found that 250 mg/day magnesium oxide for 12 weeks improved ulcer dimensions, glycemic parameters, inflammatory markers, and antioxidant capacity. A second trial reported benefits with magnesium oxide plus vitamin E [101,102].	Encouraging but preliminary clinical evidence. Small trials support further investigation; magnesium should remain an adjunct to standard DFU care.
Pressure ulcers	Potential support of cellular proliferation, protein synthesis, angiogenesis, collagen synthesis, and tissue repair.	Evidence specific to magnesium is very limited. A Cochrane review found very uncertain evidence regarding calcium, magnesium, and vitamin D supplementation for pressure-ulcer prevention [104,105].	Insufficient evidence. Routine magnesium supplementation specifically for prevention or healing is not currently supported.
Hair disorders	Potential contribution to follicular metabolism and normal hair growth; hair follicles may facilitate topical magnesium penetration.	A meta-analysis of 10 studies involving 764 participants found no significant difference in serum magnesium between patients with alopecia areata and controls. Improvements reported with nutritional supplements cannot be attributed specifically to magnesium [107,108,109,110].	Insufficient evidence. Magnesium deficiency is not an established cause of alopecia, and supplementation is not a proven treatment for hair loss.
Nail disorders	Potential contribution to nail mineral composition and growth.	Magnesium is present in the nail plate, but evidence linking its deficiency to nail abnormalities is limited. Small studies of combination supplements cannot establish magnesium’s independent effects [111,112,113,114].	Insufficient evidence. Magnesium supplementation specifically for brittle nails, onycholysis, or nail dystrophy is not currently supported.
Autoimmune skin diseases, including psoriasis and systemic lupus erythematosus (SLE)	Potential immunomodulatory and anti-inflammatory effects; modulation of cytokine production, immune-cell activity, and immune tolerance.	Observational studies associate magnesium abnormalities with inflammatory skin disorders. In a murine lupus model, a high-magnesium diet reduced skin lesions and pathogenic autoantibodies and increased regulatory T cells [29,115].	Predominantly mechanistic and preclinical evidence. Animal findings cannot be directly extrapolated to humans, and magnesium is not an established treatment for autoimmune skin diseases.

Abbreviations: DFU: diabetic foot ulcer; SLE: systemic lupus erythematosus; TEWL: transepidermal water loss; UVB: ultraviolet B.

## Data Availability

No new data were created or analyzed in this study.

## Abbreviations

The following abbreviations are used in this manuscript:

AA	alopecia areata
AD	atopic dermatitis
anti-dsDNA	anti-double-stranded DNA
ATP	adenosine triphosphate
Ca^2+^	calcium
CaCl_2_	calcium chloride
cAMP	cyclic adenosine monophosphate
COL1A1	Collagen Type I Alpha 1 Chain
COL3A1	Collagen Type III Alpha 1 Chain
CREB	cAMP Response Element-Binding Protein
CRP	C-reactive protein
CSU	chronic spontaneous urticaria
CU	Chronic urticaria
DFU	diabetic foot ulcer
DNA	deoxyribonucleic acid
ECM	extracellular-matrix
ERK	Extracellular signal-regulated kinase
FMD	flow-mediated dilation
GSK3	Glycogen Synthase Kinase-3
HLA-DR	Human Leukocyte Antigen–DR isotype
IL	interleukin
MEK	Mitogen-Activated Protein Kinase
MgCl_2_	magnesium chloride
MMP-7	Matrix Metalloproteinase-7
NF-κB	nuclear factor-κB
ROS	reactive oxygen species
TEWL	transepidermal water loss
TNF-α	Tumor Necrosis Factor alpha
TRPM	transient receptor potential melastatin channel
UV	ultraviolet
VCAM-1	Vascular Cell Adhesion Molecule 1
IFCC	International Federation of Clinical Chemistry and Laboratory Medicine
iMg	ionized magnesium
Mg^2+^	magnesium
Mg	magnesium
RBC	red blood cells
Ref.	references

## References

[1] de Baaij J.H., Hoenderop J.G., Bindels R.J. (2015). Magnesium in man: Implications for health and disease. Physiol. Rev..

[2] Grober U., Schmidt J., Kisters K. (2015). Magnesium in Prevention and Therapy. Nutrients.

[3] Dominguez L.J., Veronese N., Barbagallo M. (2024). Magnesium and the Hallmarks of Aging. Nutrients.

[4] Barbagallo M., Belvedere M., Dominguez L.J. (2009). Magnesium homeostasis and aging. Magnes. Res..

[5] Adomako E.A., Yu A.S.L. (2024). Magnesium Disorders: Core Curriculum 2024. Am. J. Kidney Dis..

[6] Ford E.S., Mokdad A.H. (2003). Dietary magnesium intake in a national sample of US adults. J. Nutr..

[7] Razzaque M.S. (2018). Magnesium: Are We Consuming Enough?. Nutrients.

[8] Huang C.W., Wen C.Y., Tsai A.P., Wang B., Tsui K.H., Hsu Y.J., Li C.J. (2026). Magnesium as a Bioenergetic Checkpoint Linking Mitochondrial Function, Metabolic Disease, and Aging. Aging Cell.

[9] Workinger J.L., Doyle R.P., Bortz J. (2018). Challenges in the Diagnosis of Magnesium Status. Nutrients.

[10] Barbagallo M., Veronese N., Dominguez L.J. (2021). Magnesium in Aging, Health and Diseases. Nutrients.

[11] Rosanoff A., Weaver C.M., Rude R.K. (2012). Suboptimal magnesium status in the United States: Are the health consequences underestimated?. Nutr. Rev..

[12] Winrich E.J., Tiwari H., Gala K.S., Royer A.J., Parajuli D., Vatsalya V. (2023). Characterization of Hypomagnesemia in Alcoholic Hepatitis Patients and Its Association with Liver Injury and Severity Markers. J. Clin. Med..

[13] Oost L.J., Tack C.J., de Baaij J.H.F. (2023). Hypomagnesemia and Cardiovascular Risk in Type 2 Diabetes. Endocr. Rev..

[14] Ye L., Zhang C., Duan Q., Shao Y., Zhou J. (2023). Association of Magnesium Depletion Score with Cardiovascular Disease and Its Association with Longitudinal Mortality in Patients with Cardiovascular Disease. J. Am. Heart Assoc..

[15] Nielsen F.H. (2018). Magnesium deficiency and increased inflammation: Current perspectives. J. Inflamm. Res..

[16] Dominguez L.J., Veronese N., Guerrero-Romero F., Barbagallo M. (2021). Magnesium in Infectious Diseases in Older People. Nutrients.

[17] Veronese N., Pizzol D., Smith L., Dominguez L.J., Barbagallo M. (2022). Effect of Magnesium Supplementation on Inflammatory Parameters: A Meta-Analysis of Randomized Controlled Trials. Nutrients.

[18] Maier J.A., Castiglioni S., Locatelli L., Zocchi M., Mazur A. (2021). Magnesium and inflammation: Advances and perspectives. Semin Cell Dev. Biol..

[19] Tennenbaum T., Yuspa S.H., Kapitulnik J. (1990). Magnesium and phosphate enrichment of culture medium stimulates the proliferation of epidermal cells from newborn and adult mice. J. Cell Physiol..

[20] Yoshino Y., Teruya T., Miyamoto C., Hirose M., Endo S., Ikari A. (2024). Unraveling the Mechanisms Involved in the Beneficial Effects of Magnesium Treatment on Skin Wound Healing. Int. J. Mol. Sci..

[21] Polefka T.G., Bianchini R.J., Shapiro S. (2012). Interaction of mineral salts with the skin: A literature survey. Int. J. Cosmet. Sci..

[22] Yang F., Xue Y., Wang F., Guo D., He Y., Zhao X., Yan F., Xu Y., Xia D., Liu Y. (2023). Sustained release of magnesium and zinc ions synergistically accelerates wound healing. Bioact. Mater..

[23] Rahmanian E., Tanideh N., Karbalay-Doust S., Mehrabani D., Rezazadeh D., Ketabchi D., EskandariRoozbahani N., Hamidizadeh N., Rahmanian F., Namazi M.R. (2024). The effect of topical magnesium on healing of pre-clinical burn wounds. Burns.

[24] Romani A.M. (2011). Cellular magnesium homeostasis. Arch. Biochem Biophys..

[25] Liu M., Dudley S.C. (2020). Magnesium, Oxidative Stress, Inflammation, and Cardiovascular Disease. Antioxidants.

[26] Fujita K., Shindo Y., Katsuta Y., Goto M., Hotta K., Oka K. (2023). Intracellular Mg(2+) protects mitochondria from oxidative stress in human keratinocytes. Commun. Biol..

[27] Shu S., Kobayashi M., Marunaka K., Yoshino Y., Goto M., Katsuta Y., Ikari A. (2022). Magnesium Supplementation Attenuates Ultraviolet-B-Induced Damage Mediated through Elevation of Polyamine Production in Human HaCaT Keratinocytes. Cells.

[28] Toyran M., Kaymak M., Vezir E., Harmanci K., Kaya A., Ginis T., Kose G., Kocabas C.N. (2012). Trace element levels in children with atopic dermatitis. J. Investig. Allergol. Clin. Immunol..

[29] Chalupczak N., Podolsky A.S., Lipner S.R. (2026). Magnesium dysregulation and risk of inflammatory skin disease: A multicenter cohort study. Dermatol. Online J..

[30] Basavaraj K.H., Darshan M.S., Shanmugavelu P., Rashmi R., Mhatre A.Y., Dhanabal S.P., Rao K.S. (2009). Study on the levels of trace elements in mild and severe psoriasis. Clin. Chim. Acta.

[31] Keskek S.O., Kirim S., Karaca A., Saler T. (2013). Low serum magnesium levels and diabetic foot ulcers. Pak. J. Med. Sci..

[32] Kurian S.J., Baral T., Unnikrishnan M.K., Benson R., Munisamy M., Saravu K., Rodrigues G.S., Rao M., Kumar A., Miraj S.S. (2023). The association between micronutrient levels and diabetic foot ulcer: A systematic review with meta-analysis. Front Endocrinol..

[33] Proksch E., Nissen H.P., Bremgartner M., Urquhart C. (2005). Bathing in a magnesium-rich Dead Sea salt solution improves skin barrier function, enhances skin hydration, and reduces inflammation in atopic dry skin. Int. J. Dermatol..

[34] Koppes S.A., Charles F., Lammers L., Frings-Dresen M., Kezic S., Rustemeyer T. (2016). Efficacy of a Cream Containing Ceramides and Magnesium in the Treatment of Mild to Moderate Atopic Dermatitis: A Randomized, Double-blind, Emollient- and Hydrocortisone-controlled Trial. Acta Derm. Venereol..

[35] Veronese N., Dominguez L.J., Pizzol D., Demurtas J., Smith L., Barbagallo M. (2021). Oral Magnesium Supplementation for Treating Glucose Metabolism Parameters in People with or at Risk of Diabetes: A Systematic Review and Meta-Analysis of Double-Blind Randomized Controlled Trials. Nutrients.

[36] Barbagallo M., Veronese N., Dominguez L.J. (2022). Magnesium in Type 2 Diabetes Mellitus, Obesity, and Metabolic Syndrome. Nutrients.

[37] Caspi R., Altman T., Dreher K., Fulcher C.A., Subhraveti P., Keseler I.M., Kothari A., Krummenacker M., Latendresse M., Mueller L.A. (2012). The MetaCyc database of metabolic pathways and enzymes and the BioCyc collection of pathway/genome databases. Nucleic Acids Res..

[38] Saris N.E., Mervaala E., Karppanen H., Khawaja J.A., Lewenstam A. (2000). Magnesium. An update on physiological, clinical and analytical aspects. Clin. Chim. Acta.

[39] Barbagallo M., Gupta R.K., Dominguez L.J., Resnick L.M. (2000). Cellular ionic alterations with age: Relation to hypertension and diabetes. J. Am. Geriatr. Soc..

[40] Barbagallo M., Dominguez L.J. (2007). Magnesium metabolism in type 2 diabetes mellitus, metabolic syndrome and insulin resistance. Arch. Biochem Biophys..

[41] Barbagallo M., Dominguez L.J., Galioto A., Ferlisi A., Cani C., Malfa L., Pineo A., Busardo A., Paolisso G. (2003). Role of magnesium in insulin action, diabetes and cardio-metabolic syndrome X. Mol. Asp. Med..

[42] Blaine J., Chonchol M., Levi M. (2015). Renal control of calcium, phosphate, and magnesium homeostasis. Clin. J. Am. Soc. Nephrol..

[43] Papagiannidou A., Mitropoulou M., Papantzikos K., Petropoulou D., Tsilingiris D., Magkos F., Dalamaga M. (2026). Hypomagnesemia: A Clinical and Nutritional Update. Curr. Nutr. Rep..

[44] Rondon L.J., Marin R. (2026). Biomarkers for assessing magnesium status. Adv. Clin. Chem..

[45] Barbagallo M., Di Bella G., Brucato V., D’Angelo D., Damiani P., Monteverde A., Belvedere M., Dominguez L.J. (2014). Serum ionized magnesium in diabetic older persons. Metabolism.

[46] Ab Rahim S.N., Nordin N., Wan Omar W.F.A., Zulkarnain S., Kumar S., Sinha S., Haque M. (2023). The Laboratory and Clinical Perspectives of Magnesium Imbalance. Cureus.

[47] Rotondi S., Mazzaferro S. (2023). Magnesium: Extracellular, intracellular or total magnesium status?. Nephrol. Dial. Transpl..

[48] Costello R.B., Nielsen F. (2017). Interpreting magnesium status to enhance clinical care: Key indicators. Curr. Opin. Clin. Nutr. Metab. Care.

[49] Witkowski M., Hubert J., Mazur A. (2011). Methods of assessment of magnesium status in humans: A systematic review. Magnes. Res..

[50] Rayana M.C., Burnett R.W., Covington A.K., D’Orazio P., Fogh-Andersen N., Jacobs E., Kulpmann W.R., Kuwa K., Larsson L., Lewenstam A. (2005). Guidelines for sampling, measuring and reporting ionized magnesium in undiluted serum, plasma or blood: International Federation of Clinical Chemistry and Laboratory Medicine (IFCC): IFCC Scientific Division, Committee on Point of Care Testing. Clin. Chem. Lab Med..

[51] Arnaud M.J. (2008). Update on the assessment of magnesium status. Br. J. Nutr..

[52] Elin R.J. (1991). Laboratory tests for the assessment of magnesium status in humans. Magnes. Trace Elem..

[53] Haftek M., Abdayem R., Guyonnet-Debersac P. (2022). Skin Minerals: Key Roles of Inorganic Elements in Skin Physiological Functions. Int. J. Mol. Sci..

[54] Hirakata Kazushi H.M., Hideyuki I., Akihiko U., Sei-Ichiro M. (2025). Modulation of keratinocyte proliferation, migration, and differentiation by calcium and magnesium. J. Cutan. Immunol. Allergy.

[55] Geesin J.C., Gordon J.S., Berg R.A. (1993). Regulation of collagen synthesis in human dermal fibroblasts by the sodium and magnesium salts of ascorbyl-2-phosphate. Skin. Pharmacol..

[56] Boraldi F., Bartolomeo A., Annovi G., Debret R., Quaglino D. (2016). Magnesium Modifies the Structural Features of Enzymatically Mineralized Collagen Gels Affecting the Retraction Capabilities of Human Dermal Fibroblasts Embedded within This 3D System. Materials.

[57] Chalupczak N.V. (2025). The role of magnesium in dermatology. JAAD Rev..

[58] Schempp C.M., Dittmar H.C., Hummler D., Simon-Haarhaus B., Schulte-Monting J., Schopf E., Simon J.C. (2000). Magnesium ions inhibit the antigen-presenting function of human epidermal Langerhans cells in vivo and in vitro. Involvement of ATPase, HLA-DR, B7 molecules, and cytokines. J. Invest Dermatol..

[59] Zheltova A.A., Kharitonova M.V., Iezhitsa I.N., Spasov A.A. (2016). Magnesium deficiency and oxidative stress: An update. Biomedicine.

[60] Chandrasekaran N.C., Weir C., Alfraji S., Grice J., Roberts M.S., Barnard R.T. (2014). Effects of magnesium deficiency--more than skin deep. Exp. Biol. Med..

[61] Cepeda V., Rodenas-Munar M., Garcia S., Bouzas C., Tur J.A. (2025). Unlocking the Power of Magnesium: A Systematic Review and Meta-Analysis Regarding Its Role in Oxidative Stress and Inflammation. Antioxidants.

[62] Lee S.E., Lee S.H. (2018). Skin Barrier and Calcium. Ann. Dermatol..

[63] Bernardini D., Nasulewic A., Mazur A., Maier J.A. (2005). Magnesium and microvascular endothelial cells: A role in inflammation and angiogenesis. Front Biosci..

[64] Dominguez L., Veronese N., Barbagallo M. (2020). Magnesium and Hypertension in Old Age. Nutrients.

[65] Teragawa H., Matsuura H., Chayama K., Oshima T. (2002). Mechanisms responsible for vasodilation upon magnesium infusion in vivo: Clinical evidence. Magnes. Res..

[66] Darooghegi Mofrad M., Djafarian K., Mozaffari H., Shab-Bidar S. (2018). Effect of magnesium supplementation on endothelial function: A systematic review and meta-analysis of randomized controlled trials. Atherosclerosis.

[67] Marques B., Klein M., da Cunha M.R., de Souza Mattos S., de Paula Nogueira L., de Paula T., Correa F.M., Oigman W., Neves M.F. (2020). Effects of Oral Magnesium Supplementation on Vascular Function: A Systematic Review and Meta-analysis of Randomized Controlled Trials. High Blood Press Cardiovasc Prev..

[68] Joris P.J., Plat J., Bakker S.J., Mensink R.P. (2017). Effects of long-term magnesium supplementation on endothelial function and cardiometabolic risk markers: A randomized controlled trial in overweight/obese adults. Sci. Rep..

[69] Elsholz F., Harteneck C., Muller W., Friedland K. (2014). Calcium—A central regulator of keratinocyte differentiation in health and disease. Eur. J. Dermatol..

[70] Denda M., Katagiri C., Hirao T., Maruyama N., Takahashi M. (1999). Some magnesium salts and a mixture of magnesium and calcium salts accelerate skin barrier recovery. Arch. Dermatol. Res..

[71] Sanabani S.S. (2026). The skin microbiome: From historical ecology to therapeutic frontiers. An. Bras. Dermatol..

[72] Amagai Y., Makita Y., Takai M., Muko R., Matsuda H., Tanaka A. (2018). Reduction in the colonization of Staphylococcus aureus on the skin surface under calcium-/magnesium-depleted conditions. Lett. Appl. Microbiol..

[73] Brandwein M., Fuks G., Israel A., Sabbah F., Hodak E., Szitenberg A., Harari M., Steinberg D., Bentwich Z., Shental N. (2019). Skin Microbiome Compositional Changes in Atopic Dermatitis Accompany Dead Sea Climatotherapy. Photochem Photobiol..

[74] Dominguez L.J., Farruggia M., Veronese N., Barbagallo M. (2021). Vitamin D Sources, Metabolism, and Deficiency: Available Compounds and Guidelines for Its Treatment. Metabolites.

[75] Wilchowski S.M., Lareau T. (2022). Psoriasis: Are Your Patients D-pleted? A Brief Literature Review on Vitamin D Deficiency and Its Role in Psoriasis. J. Clin. Aesthet. Dermatol..

[76] Zuberbier T., Abdul Latiff A.H., Abuzakouk M., Aquilina S., Asero R., Baker D., Ballmer-Weber B., Bangert C., Ben-Shoshan M., Bernstein J.A. (2022). The international EAACI/GA(2)LEN/EuroGuiDerm/APAAACI guideline for the definition, classification, diagnosis, and management of urticaria. Allergy.

[77] Barbagallo M., Dominguez L.J. (2010). Magnesium and aging. Curr. Pharm. Des..

[78] Masaki H. (2010). Role of antioxidants in the skin: Anti-aging effects. J. Dermatol. Sci..

[79] Jung Y.R., Lee E.K., Kim D.H., Park C.H., Park M.H., Jeong H.O., Yokozawa T., Tanaka T., Im D.S., Kim N.D. (2015). Upregulation of Collagen Expression via PPARbeta/delta Activation in Aged Skin by Magnesium Lithospermate B from Salvia miltiorrhiza. J. Nat. Prod..

[80] Campos P.M., de Camargo Junior F.B., de Andrade J.P., Gaspar L.R. (2012). Efficacy of cosmetic formulations containing dispersion of liposome with magnesium ascorbyl phosphate, alpha-lipoic acid and kinetin. Photochem Photobiol..

[81] Qi L., Yang X. (2025). Association between magnesium depletion score and prevalence and all-cause mortality of psoriasis among the US population. Front Nutr..

[82] Kirmit A., Kader S., Aksoy M., Bal C., Nural C., Aslan O. (2020). Trace elements and oxidative stress status in patients with psoriasis. Postep. Dermatol. Alergol..

[83] Patra P., Harrison T. (2024). Trace elements in psoriasis presentation and treatment. Arch. Dermatol. Res..

[84] Borroni G., Brazzelli V., Fornara L., Rosso R., Paulli M., Tinelli C., Ciocca O. (2013). Clinical, pathological and immunohistochemical effects of arsenical-ferruginous spa waters on mild-to-moderate psoriatic lesions: A randomized placebo-controlled study. Int. J. Immunopathol. Pharmacol..

[85] Muzumdar S., Rothe M.J. (2022). Nutrition and psoriasis. Clin. Dermatol..

[86] Saleh Bom A.Z., Majid A.Y. (2011). Serum Trace Elements (Zinc, Copper and Magnesium) Status in Iraqi Patients with Acne Vulgaris: (Case- Controlled Study). Iraqi J. Pharm. Sci..

[87] Parveen A., Ali S. (2023). A role of zinc and magnesium in the pathogenesis and severity of acne vulgaris. J. Cardiovasc. Dis. Res..

[88] Metin M.S., Kolukirik T.T., Akogul I., Metin S., Ozkoca Z. (2026). Exploring the Link Between Serum Magnesium Levels and Acne Vulgaris Severity. Selcuk Med. J..

[89] Bae J.H., Noh K.H., Jang H., Shim D., Oh J., Park S.J., Lee J.H. (2023). Clinical Benefit and Safety of Microneedle Patches Composed of Magnesium: A Pilot Study in Acne Patients. Dermatol. Ther..

[90] de Souza Pereira R. (2023). Treatment of Resistant Acne Vulgaris in Adolescents Using Dietary Supplementation with Magnesium, Phosphate and Fatty Acids (Omega 6 and 7): Comparison with 13-Cis-Retinoic Acid. J. Diet. Suppl..

[91] Zhang Y., Zhang X., Dong J., Liu F. (2025). Genetic Association Between Serum Calcium, Potassium Levels, and Rosacea: Evidence from a Two-Sample Mendelian Randomization Study. Clin. Cosmet. Investig. Dermatol..

[92] Algarin Y.A., Pulumati A., Jaalouk D., Tan J., Nouri K. (2024). The role of vitamins and nutrients in rosacea. Arch. Dermatol. Res..

[93] Dominguez L.J., Barbagallo M., Di Lorenzo G., Drago A., Scola S., Morici G., Caruso C. (1998). Bronchial reactivity and intracellular magnesium: A possible mechanism for the bronchodilating effects of magnesium in asthma. Clin. Sci. (Lond).

[94] Britton J., Pavord I., Richards K., Wisniewski A., Knox A., Lewis S., Tattersfield A., Weiss S. (1994). Dietary magnesium, lung function, wheezing, and airway hyperreactivity in a random adult population sample. Lancet.

[95] Jones L.A., Goodacre S. (2009). Magnesium sulphate in the treatment of acute asthma: Evaluation of current practice in adult emergency departments. Emerg. Med. J..

[96] Liang R.Y., Wu W., Huang J., Jiang S.P., Lin Y. (2012). Magnesium affects the cytokine secretion of CD4(+) T lymphocytes in acute asthma. J. Asthma.

[97] Stanescu C., Chiscop I., Mihalache D., Boev M., Tamas C., Stoleriu G. (2025). The Roles of Micronutrition and Nutraceuticals in Enhancing Wound Healing and Tissue Regeneration: A Systematic Review. Molecules.

[98] Pan S.C., Huang Y.J., Wang C.H., Hsu C.K., Yeh M.L. (2024). Novel Magnesium- and Silver-Loaded Dressing Promotes Tissue Regeneration in Cutaneous Wounds. Int. J. Mol. Sci..

[99] Kim Y.J., Song Y., Choe G., Yoo J., Choi J., Jung Y. (2025). Magnesium-Loaded Collagen/Hyaluronic Acid-Catechol Hydrogel Cross-Linked by Visible Light for Infected Wound Healing. ACS Appl. Bio Mater..

[100] Afzali H., Jafari Kashi A.H., Momen-Heravi M., Razzaghi R., Amirani E., Bahmani F., Gilasi H.R., Asemi Z. (2019). The effects of magnesium and vitamin E co-supplementation on wound healing and metabolic status in patients with diabetic foot ulcer: A randomized, double-blind, placebo-controlled trial. Wound Repair Regen..

[101] Razzaghi R., Pidar F., Momen-Heravi M., Bahmani F., Akbari H., Asemi Z. (2018). Magnesium Supplementation and the Effects on Wound Healing and Metabolic Status in Patients with Diabetic Foot Ulcer: A Randomized, Double-Blind, Placebo-Controlled Trial. Biol. Trace Elem. Res..

[102] Barbagallo M., Dominguez L.J. (2015). Magnesium and type 2 diabetes. World J. Diabetes.

[103] Moore Z.E., Corcoran M.A., Patton D. (2020). Nutritional interventions for treating foot ulcers in people with diabetes. Cochrane Database Syst. Rev..

[104] Munoz N., Posthauer M.E. (2022). Nutrition strategies for pressure injury management: Implementing the 2019 International Clinical Practice Guideline. Nutr. Clin. Pract..

[105] Langer G., Wan C.S., Fink A., Schwingshackl L., Schoberer D. (2024). Nutritional interventions for preventing and treating pressure ulcers. Cochrane Database Syst. Rev..

[106] Finner A.M. (2013). Nutrition and hair: Deficiencies and supplements. Dermatol. Clin..

[107] Bhat Y.J., Manzoor S., Khan A.R., Qayoom S. (2009). Trace element levels in alopecia areata. Indian J. Dermatol. Venereol. Leprol..

[108] Jin W., Zheng H., Shan B., Wu Y. (2017). Changes of serum trace elements level in patients with alopecia areata: A meta-analysis. J. Dermatol..

[109] Sant’Anna Addor F.A., Donato L.C., Melo C.S.A. (2018). Comparative evaluation between two nutritional supplements in the improvement of telogen effluvium. Clin. Cosmet. Investig. Dermatol..

[110] Chandrasekaran N.C., Sanchez W.Y., Mohammed Y.H., Grice J.E., Roberts M.S., Barnard R.T. (2016). Permeation of topically applied Magnesium ions through human skin is facilitated by hair follicles. Magnes. Res..

[111] Cashman M.W., Sloan S.B. (2010). Nutrition and nail disease. Clin. Dermatol..

[112] Chessa M.A., Iorizzo M., Richert B., Lopez-Estebaranz J.L., Rigopoulos D., Tosti A., Gupta A.K., Di Chiacchio N., Di Chiacchio N.G., Rubin A.I. (2020). Pathogenesis, Clinical Signs and Treatment Recommendations in Brittle Nails: A Review. Dermatol. Ther..

[113] Muddasani S., Lin G., Hooper J., Sloan S.B. (2021). Nutrition and nail disease. Clin. Dermatol..

[114] Curtis K.L., Lipner S.R. (2024). Vitamins for the Management of Nail Disease: A Literature Review. Skin. Appendage Disord..

[115] Verlato A., Laragione T., Bin S., Kim R.H., Salem F., Gulko P.S., Cravedi P. (2024). Revised version with tracked changes oral Magnesium reduces levels of pathogenic autoantibodies and skin disease in murine lupus. BMC Immunol..

[116] Murakami M., Yamamoto K., Taketomi Y. (2018). Phospholipase A(2) in skin biology: New insights from gene-manipulated mice and lipidomics. Inflamm. Regen..

[117] Yamamoto K., Miki Y., Sato M., Taketomi Y., Nishito Y., Taya C., Muramatsu K., Ikeda K., Nakanishi H., Taguchi R. (2015). The role of group IIF-secreted phospholipase A2 in epidermal homeostasis and hyperplasia. J. Exp. Med..

[118] Namazi M.R., Nozari F., Jafari P. (2019). Serum magnesium concentrations in vitiligo patients. Magnes. Res..

[119] Rylander R. (2014). Bioavailability of Magnesium Salts—A Review. J. Pharm. Nutr. Sci..

[120] Blancquaert L., Vervaet C., Derave W. (2019). Predicting and Testing Bioavailability of Magnesium Supplements. Nutrients.

[121] Pardo M.R., Garicano Vilar E., San Mauro Martin I., Camina Martin M.A. (2021). Bioavailability of magnesium food supplements: A systematic review. Nutrition.

[122] Ates M., Kizildag S., Yuksel O., Hosgorler F., Yuce Z., Guvendi G., Kandis S., Karakilic A., Koc B., Uysal N. (2019). Dose-Dependent Absorption Profile of Different Magnesium Compounds. Biol. Trace Elem. Res..

[123] Schuette S.A., Lashner B.A., Janghorbani M. (1994). Bioavailability of magnesium diglycinate vs magnesium oxide in patients with ileal resection. JPEN J. Parenter. Enter. Nutr..

[124] Fine K.D., Santa Ana C.A., Porter J.L., Fordtran J.S. (1991). Intestinal absorption of magnesium from food and supplements. J. Clin. Investig..

[125] Pajuelo D., Meissner J.M., Negra T., Connolly A., Mullor J.L. (2024). Comparative Clinical Study on Magnesium Absorption and Side Effects After Oral Intake of Microencapsulated Magnesium (MAGSHAPE(TM) Microcapsules) Versus Other Magnesium Sources. Nutrients.

[126] Tinsley G.M., Harty P.S., Stratton M.T., Siedler M.R., Rodriguez C. (2022). Liposomal Mineral Absorption: A Randomized Crossover Trial. Nutrients.

[127] Firoz M., Graber M. (2001). Bioavailability of US commercial magnesium preparations. Magnes. Res..

[128] Walker A.F., Marakis G., Christie S., Byng M. (2003). Mg citrate found more bioavailable than other Mg preparations in a randomised, double-blind study. Magnes. Res..

[129] National Institutes of Health (NIH) Office of Dietary Supplements (ODS) Magnesium: Fact Sheet for Health Professionals. https://ods.od.nih.gov/api/index.aspx?resourcename=Magnesium&readinglevel=Health%20Professional&outputformat=HTML.

[130] Ranade V.V., Somberg J.C. (2001). Bioavailability and pharmacokinetics of magnesium after administration of magnesium salts to humans. Am. J. Ther..

[131] Camili V., Sturup S., Heinz A. (2026). Does magnesium permeate the skin? An ex vivo investigation of magnesium ion delivery through intact and barrier-disrupted skin. Eur. J. Pharm. Biopharm..

